# Weighted Gene Co-expression Network Analysis Identifies Critical Genes for the Production of Cellulase and Xylanase in *Penicillium oxalicum*

**DOI:** 10.3389/fmicb.2020.00520

**Published:** 2020-03-27

**Authors:** Cheng-Xi Li, Shuai Zhao, Xue-Mei Luo, Jia-Xun Feng

**Affiliations:** State Key Laboratory for Conservation and Utilization of Subtropical Agro-Bioresources, Guangxi Research Center for Microbial and Enzyme Engineering Technology, College of Life Science and Technology, Guangxi University, Nanning, China

**Keywords:** co-expression network, cellulase, xylanase, time-course transcriptome, *Penicillium oxalicum*

## Abstract

Genes involved in cellular processes undergo environment-dependent co-regulation, but the co-expression patterns of fungal cellulase and xylanase-encoding genes remain unclear. Here, we identified two novel carbon sources, methylcellulose and 2-hydroxyethyl cellulose, which efficiently induced the secretion of cellulases and xylanases in *Penicillium oxalicum*. Comparative transcriptomic analyses identified carbon source-specific transcriptional patterns, mainly including major cellulase and xylanase-encoding genes, genes involved in glycolysis/gluconeogenesis and the tricarboxylic acid cycle, and genes encoding transcription factors, transporters and G protein-coupled receptors. Moreover, the weighted correlation network analysis of time-course transcriptomes, generated 17 highly connected modules. Module MEivory, comprising 120 members, included major cellulase and xylanase-encoding genes, genes encoding the key regulators PoxClrB and PoxXlnR, and a cellodextrin transporter POX06051/CdtC, which were tightly correlated with the filter-paper cellulase, carboxymethylcellulase and xylanase activities in *P. oxalicum*. An expression kinetic analysis indicated that members in MEivory were activated integrally by carbon sources, but their expressional levels were carbon source- and/or induction duration-dependent. Three uncharacterized regulatory genes in MEivory were identified, which regulate the production of cellulases and xylanases in *P. oxalicum*. These findings provide insights into the mechanisms associated with the synthesis and secretion of fungal cellulases and xylanases, and a guide for *P. oxalicum* application in biotechnology.

## Introduction

Soil microbiomes play an essential role in the cycles and balances of terrestrial ecosystems. A major population of organisms, filamentous fungi, break down organic matter to gain nutrients for survival, owing to their ability to synthesize and secrete plant-biomass degrading enzymes (PDEs; Gusakov, 2011). Natural enzyme production by fungi, however, is very low, restricting their industrial application. Although the isolation and characterization of PDEs has been performed, success has been limited, because of the diversity of PDEs, signal transduction specificity in response to carbon sources and the complex synergy of PDEs required to degrade plant biomass (Zhang and Zhang, 2013; Parameswaran et al., 2018; Schmoll, 2018; Antonieto et al., 2019; Srivastava et al., 2019). Breeding by genetic modification has been a potential strategy to improve fungal enzyme production. However, to best perform this strategy, it is necessary to understand the mechanisms responsible for PDE synthesis and secretion by fungi, specifically aspects associated with the expression of enzyme-encoding genes and their expressional regulation.

The effects of PDEs on plant-biomass degradation result from a co-expression network of genes mediated by carbon sources, including enzyme and other associated genes. Recently, the development of high-throughput sequencing technologies (Shendure et al., 2017) and their corresponding analysis tools, has resulted in decreasing sequencing costs ($0.012 per Mbp^1^), thereby facilitating large-scale analyses of omics data that can uncover universal mechanisms at the molecular level. Weighted gene co-expression network analysis (WGCNA) is a systems biology method for describing correlation patterns among genes based on RNA-sequencing data and it is widely used for the construction of gene co-expression networks and identification of critical hub genes in several biological contexts, including yeasts, mammals and plants.

High expression of fungal PDE-encoding genes requires a corresponding inducer. At present, insoluble biomass [i.e., Avicel (AV), xylan and raw starch] and soluble sugars (i.e., D-xylose, cellobiose, lactose, and sophorose) are commonly used for inducing PDE production in filamentous fungi, including *Trichoderma*, *Aspergillus*, and *Penicillium* (Amore et al., 2013; Tani et al., 2014). Several studies postulated the inducer function of low molecular weight and soluble compounds from plant biomass. When cultivated on raw insoluble biomass, fungal cells initially produce a basal level of PDEs that hydrolyze biomass to generate a soluble inducer (Amore et al., 2013). Therefore, a potent inducer is essential for high PDE yields in filamentous fungi.

Transcription of fungal PDE-encoding genes is tightly controlled by specific transcription factors (TFs). Several TFs regulating the expression of PDE-encoding genes were identified, including the cellulase activator CLR-2/ClrB (Coradetti et al., 2012; Zhao et al., 2016), xylanase activator XlnR/Xyr1/XLR-1 (Amore et al., 2013; Li et al., 2015), amylase activator AmyR (Li et al., 2015; Benocci et al., 2017) and carbon catabolite repressor CreA/CRE1/CRE-1. These TFs can directly bind to the promoters of key PDE-encoding genes to perform their regulatory roles. Remarkably, XlnR negatively regulates cellulase production, while positively regulating xylanase production in *P. oxalicum* (Li et al., 2015). Both ClrB and XlnR dynamically control the transcriptional levels of major cellulase and/or xylanase genes under the induction of cellulose, but their relative importance depends on the species (Benocci et al., 2017). AmyR functions in the opposite way, in the expressional regulation between amylase and cellulase genes in *P. oxalicum* (Li et al., 2015). CreA/CRE1/CRE-1 negatively regulates the transcripts of almost all PDE-encoding genes and their regulatory genes (Li et al., 2015; Benocci et al., 2017). This suggests that the expression of different PDE-encoding genes is co-regulated, but shows diversity. In addition, several studies indicate that expression of PDE-encoding genes is also co-regulated with that of genes involved in asexual reproduction and secondary metabolites, such as *BrlA* and *LaeA* (Qin et al., 2013; Li et al., 2016).

The soil filamentous fungus *P. oxalicum* is increasingly attracting attention as a plant biomass bio-refinery, for biofuels and other high-value-added biochemical products, because it secretes a more balanced PDE system and has a greater β-glucosidase (BGL) activity than *Trichoderma reesei* (Zhao et al., 2016; Su et al., 2017). Lignocelluloses are degraded into glucose (GLU) through the synergistic action of cellobiohydrolase (CBH), endo-β-1,4-glucanase (EG) and BGLs (Wang et al., 2013). Of these, BGL plays key roles in lignocellulosic bioconversion, directly affecting the operational cost of bio-refineries (Srivastava et al., 2019). Genomic annotation of the *P. oxalicum* wild-type strain HP7-1 revealed 477 genes encoding carbohydrate-active enzymes (CAZymes), including three CBH genes, 11 EG genes, 11 BGL genes and 11 endo-β-1,4-xylanase (XYN) genes, and 484 genes encoding putative TFs (Zhao et al., 2016).

In this study, to find an efficient inducing carbon source for *P. oxalicum*, we investigated the effects of 23 carbon sources on cellulase and xylanase production in the *P. oxalicum* strain Δ*PoxKu70*. Of these, two novel carbon sources [methylcellulose (MC) and 2-hydroxyethyl cellulose (HEC)] efficiently promoted enzyme production. Transcriptional profiling of the Δ*PoxKu70* was also performed, when cultured on AV, MC, HEC or WB. In addition, we employed WGCNA to analyze 24 time-course transcriptomes, with three biological replicates for each sample, from *P*. *oxalicum* under various culture conditions. The co-expression modules of cellulase and xylanase-encoding genes were identified and characterized in *P. oxalicum*. Moreover, the regulatory roles of modular TFs in the production of cellulase and xylanase were investigated.

## Materials and Methods

### Fungal Strains and Culture Conditions

The fungal strain used in this work is the highly efficient transformation strain Δ*PoxKu70* (#3.15650, China General Microbiological Culture Collection, CGMCC), derived from the *P. oxalicum* wild-type strain HP7-1 (CGMCC no. 10781) by deleting the gene *PoxKu70* (Zhao et al., 2016). Fresh spores of Δ*PoxKu70* were statically incubated on potato-dextrose agar plates at 28°C for 6 days, collected and resuspended in sterile water containing 0.1% Tween 80 to a final concentration of 1 × 10^8^ per mL for further study.

For RNA-sequencing, the fresh spore suspension described above (500 μL) was inoculated into GLU medium (100 mL) (Yan et al., 2017) and placed in a shaker at 180 rpm for 24 h at 28°C. The hyphae were filtered using a vacuum pump and washed once with sterile water (50 mL). Approximately 1 g of hyphae was transferred to basic medium (100 mL), separately supplemented with various carbon sources, including 1% (w/v) AV, WB, MC, HEV, and GLU, and incubated in a shaker at 180 rpm for 4–48 h at 28°C. The medium with no carbon source (NC) and GLU were used as controls. Hyphae were harvested for total RNA extraction and the remaining medium (crude enzyme solution) was used for the measurements of enzymatic activities.

### Total RNA Extraction

Total RNAs from *P. oxalicum* hyphae under different culture conditions were extracted using a TRIzol RNA Kit (Life Technologies, Carlsbad, CA, United States), as described previously (Yan et al., 2017).

### RNA-Sequencing and Data Analysis

RNA-sequencing of *P. oxalicum* total RNAs was carried out on a BGISEQ-500 platform at BGI, Shenzhen, China. In brief, total RNAs were pretreated with DNase I to eliminate double- and single-stranded DNA contaminants, and the mRNA was purified after mRNA enrichment using oligo (dT), and attached to magnetic beads. Subsequently, the purified mRNA was fragmented and used as the template for the synthesis of first-strand cDNA with random hexamer-primed reverse transcription, followed by the synthesis of the second-strand cDNA. The generated cDNA was subjected to end repair and 5′-phosphorylation, as well as the addition of a single base A and ligation with a bubble adapter. The products were enriched by PCR amplification and purified. Double-strand DNA was denatured by heating and subsequently circularized to construct a library of single-strand circular DNAs for further sequencing.

The generated raw reads were filtered using SOAPnuke v1.5.2 to obtain clean reads with parameters −l 15, −q 0.2, and −n 0.05 (Chen et al., 2018). Clean reads were mapped to the genome of *P. oxalicum* wild-type strain HP7-1 (Zhao et al., 2016) using Hierarchical Indexing for Spliced Alignment of Transcripts v2.0.4 software (Kim et al., 2015). Bowtie2 v2.2.5 (Langmead and Salzberg, 2012) was employed for gene mapping. Fragments per kilobase of exon per million mapped reads (FPKM) values, representing the gene transcriptional levels, were calculated using RSEM v1.2.12 (Li and Dewey, 2011). DEGs were screened for by comparative transcriptomes using the DESeq tool (Love et al., 2014), with a cutoff of *p*-value ≤ 0.05 and | log2 fold change| ≥ 1. A heatmap of the DEGs was constructed using the R package, pheatmap^2^.

### Enzymatic Activity Measurement

Fungal cellulase and xylanase activities were measured as described previously (Yan et al., 2017).

### Measurement of Intracellular Protein

The cultivated mycelium collected from 100 mL of culture was harvested, washed and ground into powder with liquid nitrogen. Protein extraction buffer [15 mL, containing NaCl 8.5, Na_2_HPO_4_ 2.2, NaH_2_PO_4_ 0.2, phenylmethylsulfonyl fluoride 0.87 and ethylenediaminetetraacetic acid 1.86 g/L, at pH 7.4] and 0.25 mm dia. glass balls (4 g) were added to the powder. The mixture was put on ice for 5 min, shaken for 1 min and put back on ice for 1 min, repeated in triplicate. The protein was separated via centrifugation at 13000 × *g* for 10 min. Protein concentration was determined using the Pierce^TM^ detergent- compatible Bradford assay kit (Pierce Biotechnology, Rockford, IL, United States).

### Measurement of Growth Curves of *P. oxalicum*

Hyphal weight from *P. oxalicum* was quantified as total intracellular protein content per 100 mL of culture. Fresh spores (10^8^) of Δ*PoxKu70* were directly inoculated into medium (100 mL) containing MC, HEC, AV, WB, GLU, or NC, respectively. The inoculated media were cultivated at 28°C for 24–72 h. The mixture of mycelia and/or insoluble carbon sources were collected from 100 mL of culture by vacuum filtration every 12 h, and then the entire mycelia collected were used for extraction of intracellular proteins as described above. The resulting intracellular protein content data were used for construction of growth curves for *P. oxalicum*.

### WGCNA

The R package, WGCNA was used for WGCNA (Langfelder and Horvath, 2008). The average FPKM value of three biological replicates for each gene was calculated and used for further analyses. R package HCLUST^3^ was used for detecting outliers of samples with the clustering method “average.” The modules were identified using the dynamic tree cut method based on dissTOM hierarchical clustering and the other parameters, deepSplit = 2 and minModuleSize = 30. The modules having dissimilarity coefficients of 0.25 (cutHeight = 0.25) were merged to obtain final co-expression modules. The moduleTraitCor function was used to correlate (hemi-) cellulase activity traits with co-expression modules according to the tutorials of WGCNA (Langfelder and Horvath, 2016). Briefly, we used module Eigengenes from WGCNA package to process the expression profile in each module to generate the corresponding module eigengene, and subsequently related the module eigengenes to the activities of cellulases and xylanases of *P. oxalicum* strain Δ*PoxKu70* cultured on various carbon sources through calculating the correlation coefficients using moduleTraitCor. Significance analysis was carried out using corPvalureStudent. Heatmap showing the correlation coefficients and corresponding values was constructed using labeled heatmap. Values (*p*-value ≤ 0.05 and correlation coefficients ≥ 0.4) indicate correlations between samples (Langfelder and Horvath, 2008). Relevance matrix calculations and significance level calculations of correlation coefficients between global genes were performed using the R-package Hmisc^4^.

### Construction of Deletion Mutants

Deletion mutants were constructed in the *P. oxalicum* parental strain Δ*PoxKu70* according to the methods described by Yan et al. (2017). The primers used for mutant construction are listed in Supplementary Table S1.

### Availability of Data and Materials

All transcriptomic data are available in the GEO database with accession number GSE133258 on NCBI. The DNA sequences for *POX01118*, *POX01474*, and *POX01678* have been deposited in the GenBank database (accession numbers MN529555–MN529557), respectively.

## Results and Discussion

### Enzyme Activities From *P. oxalicum* Induced by Various Carbon Sources

**FIGURE 1:**
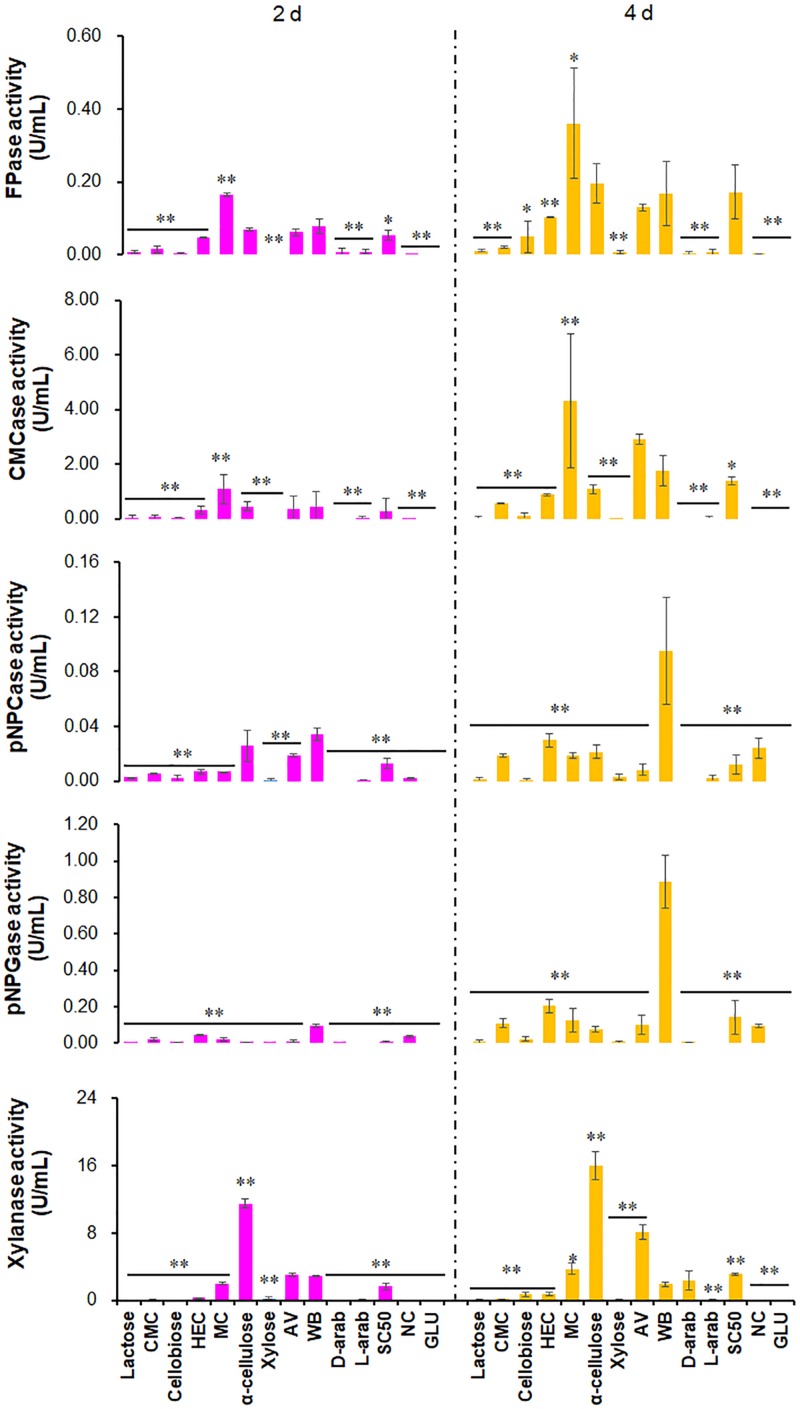
Activities of crude enzymes produced by *P. oxalicum* strain Δ*PoxKu70* cultured on various carbon sources. Crude enzymes were collected from the Δ*PoxKu70* cultivated for 2–4 days after a transfer from GLU. ^∗^, *p* ≤ 0.05; ^∗∗^, *p* ≤ 0.01 indicate the significance of differences between the results on various carbon sources and WB. CMC, carboxymethylcellulose; HEC, 2-hydroxyethyl cellulose; MC, methyl cellulose; WB, wheat bran; AV, Avicel; D-arab, D-arabinose; L-arab, L-arabinose; SC50, Sigmacell cellulose; NC, no carbon source; GLU, glucose; FPase, filter paper cellulase; CMCase, carboxymethylcellulase; pNPCase, *p*-nitrophenyl-β-cellobiosidase; pNPGase, *p*-nitrophenyl-β-glucopyranosidase.

To screen for strong inducers of cellulase activity in the Δ*PoxKu70*, 23 carbon sources were chosen (Supplementary Table S2). NC was used as a control. Activities of four cellulases [filter paper cellulase (FPase), carboxymethylcellulase (CMCase), *p*-nitrophenyl-β-cellobiosidase (pNPCase) and *p*-nitrophenyl-β-glucopyranosidase (pNPGase)], plus xylanase were measured after 2–4 days of culture after transfer from glucose. The activities of cellulases and xylanases showed diversity under the induction of different carbon sources. For example, FPase activities of Δ*PoxKu70* with more than 0.01 U/mL were detected when cultured on AV, CMC, HEC, MC, WB, α-cellulose and Sigmacell cellulose (SC50; Sigma-Aldrich, Darmstadt, Germany) for 2 days after a transfer from GLU, or on additional lactose and cellobiose (Sigma-Aldrich) for 4 days. Of these, FPase activity on MC was the highest, at 0.16 U/mL at 2 days and 0.36 U/mL at 4 days, followed by WB (0.08 U/mL at 2 days) and α-cellulose (0.20 U/mL at 4 days), respectively. CMCase activities of the Δ*PoxKu70* ranged from 0.02 to 4.31 U/mL when cultivated on lactose, CMC, cellobiose, HEC, MC, α-cellulose, AV, WB, D/L-arabinose, or Sigmacell cellulose, but they could not be detected on other carbon sources. Δ*PoxKu70* cultivated on MC possessed the highest activity of CMCase (i.e., 1.08 U/mL at 2 days and 4.31 U/mL at 4 days), followed by AV (2.91 U/mL at 4 days) and WB (1.75 U/mL at 4 days), respectively. Both pNPCase and pNPGase activities were low, ranging from 0.01 to 0.89 U/mL on lactose, CMC, cellobiose, HEC, MC, xylose, α-cellulose, AV, WB and Sigmacell cellulose. WB was the most efficient carbon source to induce pNPCase and pNPGase activities of *P. oxalicum*, among the tested carbon sources. Xylanase activity in Δ*PoxKu70* was 0.02–16.07 U/mL on 12 carbon sources, including lactose, CMC, cellobiose, HEC, MC, xylose, α-cellulose, AV, WB, D/L-arabinose and Sigmacell cellulose, except for D-arabinose at 2 days. Xylanase activity on α-cellulose was higher than that of other carbon sources. Δ*PoxKu70* hardly produced any cellulases or xylanases when cultured on NC. Overall, the inducibility of FPase and CMCase production by MC was strongest at 2–4 days. WB was the most efficient carbon source to induce pNPCase and pNPGase production and α-cellulose for xylanase production, among the tested carbon sources (*p* < 0.05; Figure 1).

Methylcellulose and HEC are novel carbon sources for induction of cellulase and xylanase production from *P. oxalicum*. Compared with the already known WB and AV, induction by MC was stronger, specifically for FPase and CMCase, while slightly lower for HEC. MC and HEC are derived from cellulose through methylation and hydroxyethylation, respectively, and are widely used in tissue engineering (Dutta et al., 2019). MC mainly induced the secretion of EGs that randomly cleave internal β-1,4-glycosidic bonds of cellulose chains to generate numbers of chain ends for CBHs, thereby resulting in increased FPase activity. By contrast, HEC stimulated all types of cellulases, including CBHs, EGs and BGLs, and increased FPase activity, dependent on a strong synergistic interaction. Remarkably, strong inducers for *Tricherdoma* and *Aspergillus*, such as lactose (Amore et al., 2013), showed low induction of cellulase and xylanase production in *P. oxalicum*, probably through repressing the expression of cellulase and xylanase-encoding genes, but this needs to be confirmed. These data also imply that different signal transduction pathways and regulation mechanisms for cellulase and xylanase-encoding genes exist in *P. oxalicum*.

### Effects on *P. oxalicum* Growth by the Five Chosen Carbon Sources Above

**FIGURE 2:**
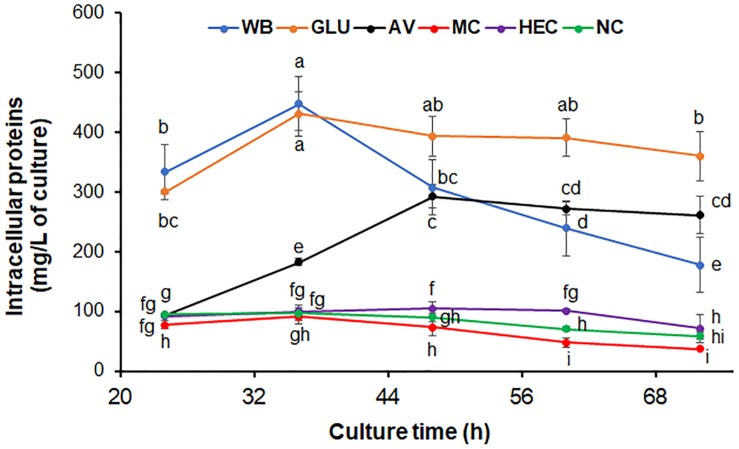
Growth curves of *P. oxalicum* strain Δ*PoxKu70*. Fresh spores of Δ*PoxKu70* were inoculated into medium containing each carbon source and cultivated for 24 to 72 h. Total intracellular proteins from all the *P. oxalicum* hyphae in the culture were extracted and measured. The lowercase letters indicate significant difference at *p* < 0.05 probability level, assessed by Student’s *t*-test. HEC, 2-hydroxyethyl cellulose; MC, methyl cellulose; WB, wheat bran; AV, Avicel; NC, no carbon source; GLU, glucose.

Five carbon sources including two novel inducers MC and HEC, commonly used inducers AV, WB and repressor GLU, were selected for further study. The growth curves of Δ*PoxKu70* were determined after direct inoculation into medium containing AV, WB, MC, HEC, and GLU, respectively, with an NC control. Here the intracellular protein contents measured from the entire mycelia collected from the inoculated media were used for construction of the growth curves. Growth of Δ*PoxKu70* cultured on GLU, WB, and AV, was faster than that on HEC, MC, and NC, except on AV before 24 h (Figure 2). Biomass accumulation of Δ*PoxKu70* on AV at 24 h was the same as that on HEC, NC, and MC. During the early period of culture (before 36 h), Δ*PoxKu70* grew similarly on WB and on GLU since WB contains some free GLU, but growth on WB was slower after 36 h, since free GLU would have been consumed and GLU then needed to be released via WB hydrolysis by cellulases or amylases. On AV, Δ*PoxKu70* grew slower than on WB before 36 h, faster after 64 h, but slower than on GLU during the whole culture time. The growth of Δ*PoxKu70* was similar, when cultured on HEC, MC or NC from 24 to 36 h, while decreased on MC and increased on HEC compared with on NC after 36 h (Figure 2).

WB is rich in polysaccharides (i.e., starch and cello-oligosaccharides), simple sugars such as GLU and crude proteins (Sun et al., 2008; Sardari et al., 2019). When cultured on WB, *P. oxalicum* initially grew on the GLU in WB, as well as it did on GLU medium. Once the GLU was exhausted, the *P. oxalicum* cells would have been forced to produce cellulases and/or amylases to degrade cello-oligosaccharides and/or starch into GLU, resulting in a growth delay (Sun et al., 2008). Avicel is a crystalline cellulose that cannot be directly utilized by fungal cells (Yang et al., 2011), but can be digested into GLU by cellulases from *P. oxalicum*. Therefore, mycelium accumulation gradually increased during the culture time, dependent on GLU release. During the early culture period, the growth of *P. oxalicum* on HEC was similar to that on NC because the inoculated hyphae had only stored energy available, but sped up after nutrients were released from HEC degradation. Surprisingly, MC inhibited fungal growth, which might be attributable to toxicity of the released methyl-glucose or oligosaccharides from MC degradation.

### Carbon Source-Specific Transcriptional Patterns of *P. oxalicum*

To further investigate carbon source-specific transcriptional patterns of *P*. *oxalicum* growth on the five carbon sources WB, HEC, AV, MC, and GLU, time-course transcriptomes from Δ*PoxKu70*, cultured at four time points (4, 12, 24, and 48 h), after a transfer from GLU, were RNA-sequenced, with NC as control. Each sample had three biological replications. A total of 72 transcriptomes was sequenced. A total of 1,548,422,794 clean reads was generated, with an average length of 50 bp per read (Supplementary Table S3), and were mapped to the genome of *P*. *oxalicum* wild-type strain HP7-1 (Zhao et al., 2016), resulting in an average 98.2% mapping rate. Correlation coefficients were high (*R* > 0.82) for the three biological replicates produced under each set of culture conditions (Supplementary Figures S1–S4). Furthermore, a principal components analysis revealed no outliers among the three biological replicates for each sample (Supplementary Figure S5), indicating that all the transcriptomes were reliable for further analyses.

**FIGURE 3:**
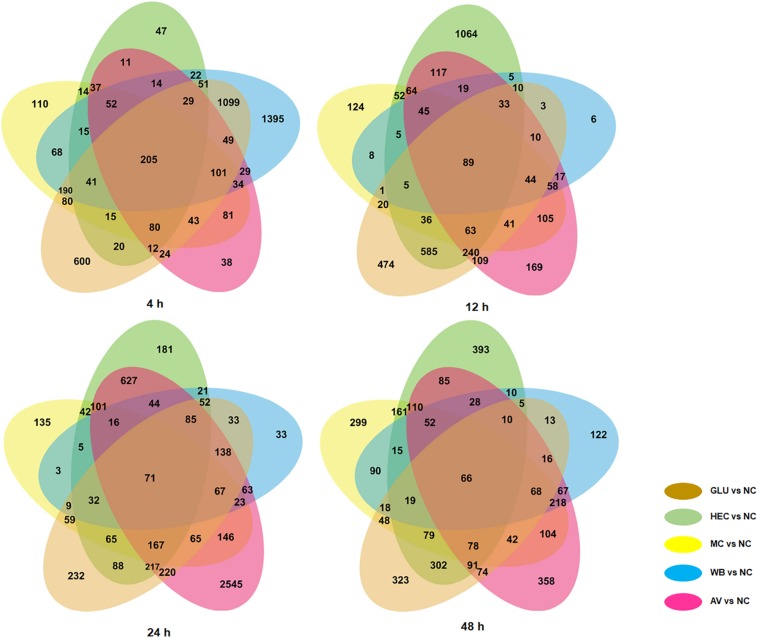
Venn diagram indicating numbers of unique and shared DEGs in transcriptomes from *P. oxalicum* strain Δ*PoxKu70* in the presence of different carbon sources (AV, MC, HEC, WB, and GLU) compared with NC. DEGs were selected using the thresholds of *p*-value ≤ 0.05 and | log2 fold change| ≥ 1. DEGs, differentially expressed genes; AV, Avicel; MC, methyl cellulose; HEC, 2-hydroxyethyl cellulose; GLU, glucose; NC, no carbon source.

We comparatively analyzed 72 time-course transcriptomes described above using DESeq software (Love et al., 2014). Using *p*-value ≤ 0.05 and | log2 fold change| ≥ 1 as thresholds, differentially expressed genes (DEGs) were screened in transcriptomes of *P. oxalicum* on five carbon sources (AV, WB, HEC, MC, and GLU) compared with on NC as the control, respectively. Each comparison (Δ*PoxKu70*_AV vs. Δ*PoxKu70*_NC, Δ*PoxKu70*_MC vs. Δ*PoxKu70*_NC, Δ*PoxKu70*_HEC vs. Δ*PoxKu70*_NC, Δ*PoxKu70*_WB vs. Δ*PoxKu70*_NC, and Δ*PoxKu70*_GLU vs. Δ*PoxKu70*_NC) shared 358–3,394 DEGs at 4–48 h. All five comparisons co-shared 205, 89, 71 and 66 DEGs at each time point, respectively (Figure 3). The Kyoto Encyclopedia of Genes and Genomes (KEGG) annotation indicated that these co-shared DEGs were mainly involved in metabolism, specifically starch and carbohydrate metabolism (Supplementary Figures S6–S9).

**FIGURE 4:**
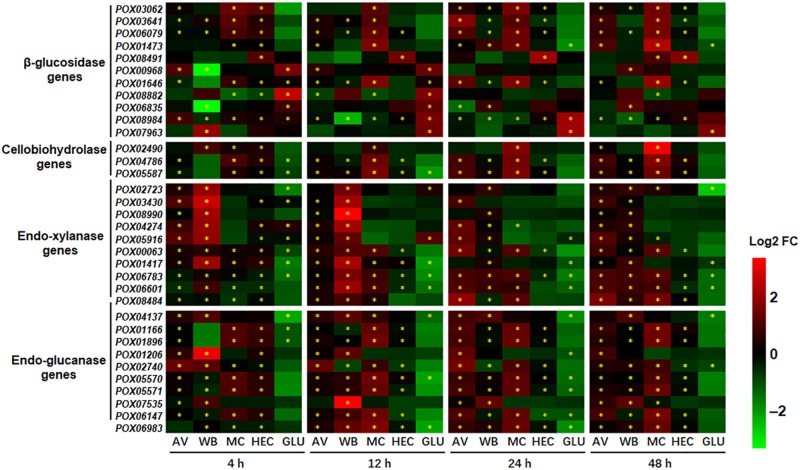
Heatmap indicating transcriptional levels of cellulase and endo-xylanase genes in *P. oxalicum* on different carbon sources (AV, MC, HEC, WB, and GLU) compared with NC. * represents the DEGs (*p* ≤ 0.05, | log2 fold change) | ≥ 1.0). The heatmap shows the log2 fold change of each gene on different carbon sources compared with that on NC. DEGs, differentially expressed genes; AV, Avicel; MC, methyl cellulose; HEC, 2-hydroxyethyl cellulose; GLU, glucose; NC, no carbon source; FC, fold change.

As expected, 16–28 of the 35 cellulase and endo-xylanase genes in the genome of HP7-1 (Zhao et al., 2016) showed significant alterations at the transcriptional level (*p* < 0.05; | log2 fold change| ≥ 1) in response to AV, WB, MC, and HEC for 4 to 48 h, compared with that on NC, including the known key cellulase and xylanase-encoding genes, such as *POX05587*/*Cel7A-2* (*cbh1*), *POX05570*/*Cel45A*, *POX06147*/*Cel5A*, *POX07535*/*Cel12A*, *POX06835*/*Bgl1*, *POX00063*/*Xyn10A*, and *POX06783*/*Xyn11A*; most of them were up-regulated. Comparative analyses indicated that the largest number of DEGs was induced by AV (26–28), followed by WB (24–27). The extent of variation induced by MC was the widest (1.01 < | log2 fold change| < 13.08), followed by WB (1.03 < | log2 fold change| < 12.39) and AV (1.01 < | log2 fold change| < 10.45). It should be noted that MC strongly induced the expression of key cellulase genes including two *cbhs* (*POX05587*/*Cel7A-2* and *POX04786/Cel6A*), four *egs* (*POX01166/Cel5B*, *POX01896*, *POX05570/Cel45A*, and *POX05571/Cel7B*), and one *bgl* (*POX06079*) compared with WB, AV and HEC (Figure 4). Transcriptional levels of most cellulase and xylanase-encoding genes exhibited an increasing trend during the induction time. Conversely, during growth on GLU, 9–17 of 35 cellulase and xylanase-encoding genes showed alteration (*p* < 0.05; | log2 fold change| ≥ 1) compared with that on NC, most of them down-regulated (Figure 4).

**FIGURE 5:**
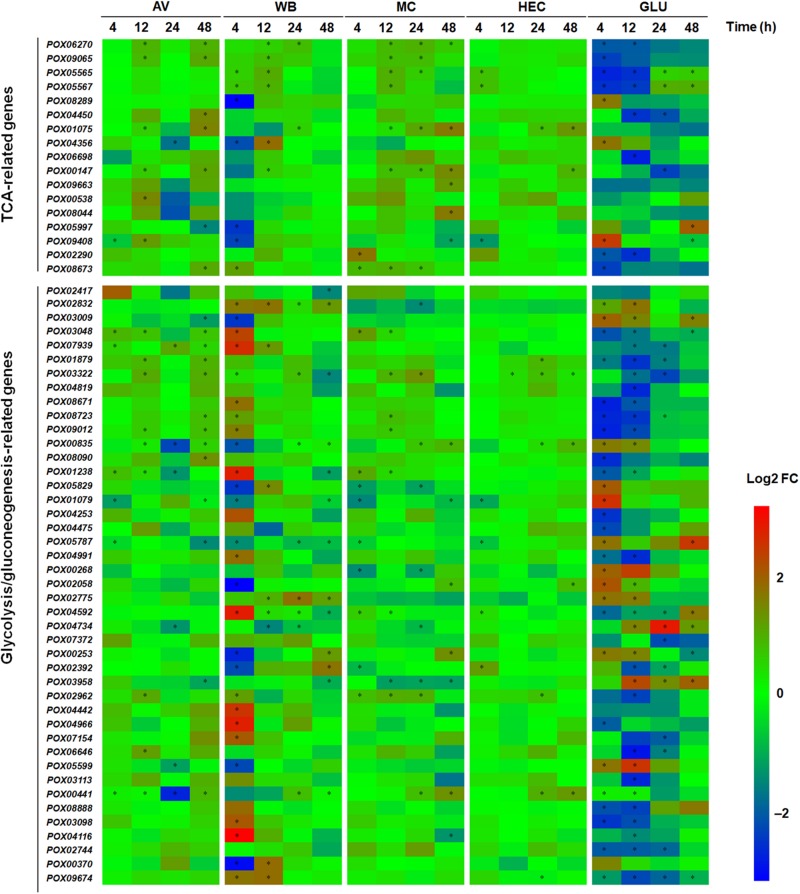
Heatmap indicating transcriptional levels of DEGs involved in glycolysis/gluconeogenesis and TCA cycle in *P. oxalicum* on different carbon sources (AV, MC, HEC, WB, and GLU) compared with NC. ^∗^ represents the DEGs (*p* ≤ 0.05, | log2 fold change) | ≥ 1.0). The heatmap shows the log2 fold change of each gene on different carbon sources compared with that on NC. DEGs: differentially expressed genes; AV, Avicel; MC, methyl cellulose; HEC, 2-hydroxyethyl cellulose; GLU, glucose; NC, no carbon source; TCA, tricarboxylic acid cycle; FC, fold change.

Carbon sources are able to induce the genes encoding proteins involved in carbon metabolism and the tricarboxylic acid (TCA) cycle (He et al., 2013). Here, comparative transcriptomic analyses indicated that 60 DEGs were annotated to be involved in glycolysis/gluconeogenesis and the TCA cycle in *P. oxalicum.* These DEGs appeared at least once, in all comparisons between the transcriptomes expressed during growth on the five carbon sources and NC. These proteins encoded by DEGs included the major rate-limiting enzymes, hexokinase POX01079, POX04253, POX04475, and POX05787; phosphofructokinase POX08888; pyruvate kinase POX02744 in glycolysis, and citrate synthases POX01075 and POX04356; isocitrate dehydrogenases POX06270 and POX09065; oxoglutarate dehydrogenases POX00538 and POX09663 in the TCA cycle (Supplementary Figure S10). Of the 60 DEGs, 7–20, 10–34, 11–15, 1–7, and 12–38 DEGs were susceptible to induction by growth on AV, WB, MC, HEC, and GLU for 4–48 h, to various degrees, respectively, compared with NC (Figure 5). These results suggested that the expression of glycolysis/gluconeogenesis- and TCA cycle-related genes are most strongly induced by GLU and WB, followed by MC and AV. Most of the DEGs involved in glycolysis/gluconeogenesis and the TCA cycle were up-regulated in response to AV, MC, WB, and HEC, especially 20 of 34 DEGs with WB at 4 h compared with NC, but generally, much less up-regulated than in response to GLU (Figure 5). Most of 60 DEGs showed active alteration during the early period (up to 12 h) of growth on WB and GLU. Surprisingly, the transcriptional levels of those DEGs encoding rate-limiting enzymes showed variance under different conditions, for example, genes *POX01079* and *POX05787* were down-regulated on AV, WB, MC, and HEC compared with NC, but up-regulated on GLU at 4 h. At 12 h, however, their transcriptional levels showed no alteration (Figure 5).

**FIGURE 6:**
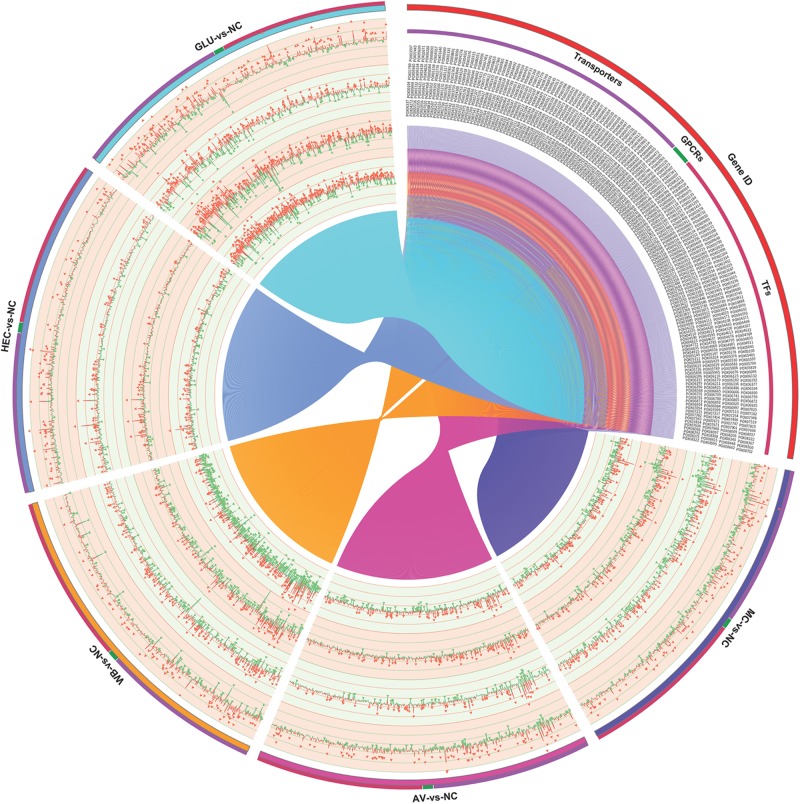
Expressional analysis of genes encoding transcription factors, transporters and G protein-coupled receptors in *P. oxalicum* on different carbon sources (AV, MC, HEC, WB, and GLU) compared with NC. AV, Avicel; MC, methyl cellulose; HEC, 2-hydroxyethyl cellulose; GLU, glucose; NC, no carbon source; TFs, transcription factors; GPCRs, G-protein coupled receptors.

The detected DEGs included other genes possibly involved in synthesis of cellulase and xylanase, such as TFs, transporters and G protein-coupled receptor (GPCR) genes. Comparative transcriptomic analyses showed that 283 DEGs encoded putative TFs. Approximately 70% of these detected TFs belong to the zinc finger family, followed by winged helix repressors (8.4%). Their transcriptional levels varied in response to the different carbon sources and the duration of induction, for instance, 44 and 165 DEGs on MC and WB at 4 h compared with that on NC, respectively, and 34 and 51 at 12 h. Screening for the top ten upregulated/downregulated TF genes for each comparison detected a total of 163, including several known regulatory genes, i.e., *POX01167/PoxCxrA*, *POX04420/PoxCxrB*, *POX01960/PoxClrB*, *POX02484*, *POX03890/PoxAmyR*, *POX08292/PoxMBF1*, and *POX06534/BrlA* (Qin et al., 2013; Li et al., 2015; Zhao et al., 2016, 2019; Yan et al., 2017; Figure 6).

A total of 271 DEGs encoding proteins were annotated as ABC transporter-like (IPR003439), sugar/inositol transporter (IPR003663), or major facilitator superfamily (IPR011701). Numbers of transporter DEGs in response to AV and MC increased, after longer induction duration compared with NC, but numbers decreased in response to GLU and WB, and hardly changed in response to HEC. Homologous analysis with known GPCR in filamentous fungi (Affeldt et al., 2014; Martín et al., 2019) detected 18 DEGs encoding GPCRs. Their expression was active under induction by WB and GLU during the early period (Figure 6). The comparative transcriptomic analysis detected many more DEGs, but most of them were not related to cellulase and xylanase production in filamentous fungi; this needs to be studied further.

### Identification of Co-expression Modules of Cellulase- and Xylanase-Encoding Genes in *P. oxalicum*

We hypothesized that the genes mediated by carbon sources would form tight modules, also called associations, with each other, in gene co-expression networks. The 72 transcriptomes obtained were subjected to WGCNA (Langfelder and Horvath, 2008). Sample outlier detection was evaluated first. The average values of FPKMs generated by three biological repeats for each sample were used as input data for a WGCNA. Sample outlier detection revealed that MC48, transcriptome from *P. oxalicum* cultured on MC for 48 h, had the highest outlier potential. The outlier potential of MC48 could be attributed to the efficient induction of gene expression by MC at 48 h, because the enzyme extracts of *P. oxalicum* showed the highest cellulase and xylanase activities, except for pNPGase (Supplementary Figure S11). Therefore, MC48 needed to be retained for further WGCNA.

**FIGURE 7:**
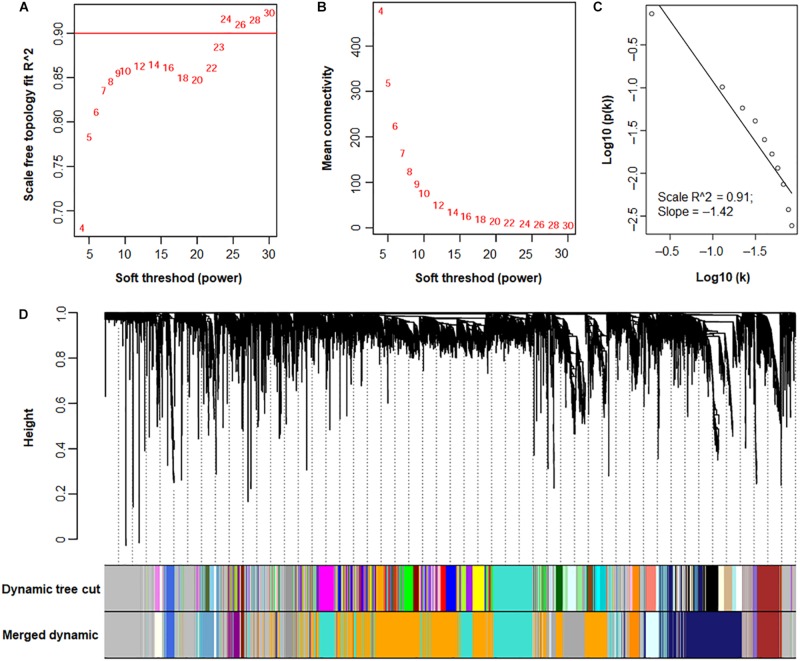
A WGCNA of transcriptomes from *P. oxalicum* under different culture conditions. **(A)** Determination of soft-power based on the adjacency matrix. **(B)** Mean connectivity. **(C)** Confirmation of scale-free topology. Based on **(A–C)**, the soft-powder was selected as 24, resulting in a scale-free gene co-expression network topology. **(D)** Identification of functional modules illustrated with various colors. Cluster dendrograms were generated using hierarchical clustering. Original modules were identified using the Dynamic Tree Cutting method, and merged modules were generated according to the correlations of modules, with module dissimilarity coefficients of ≤ 0.25.

Subsequently, the pickSoftThreshold function was used to calculate the soft thresholds. When soft-power was set at 24, the Scale Free Topology Model Fit *R*^2^ was ≥ 0.9 (Figures 7A,B). To confirm that the topology of the gene co-expression network was scale-free, soft connectivity was determined. The correlation coefficient between the log (k) of the node number with connectivity *K* and the log [p (k)] of the probability of node occurrence was ≥ 0.9 (Figure 7C), suggesting that the topology of the network trended scale-free when the soft-power was 24.

**FIGURE 8:**
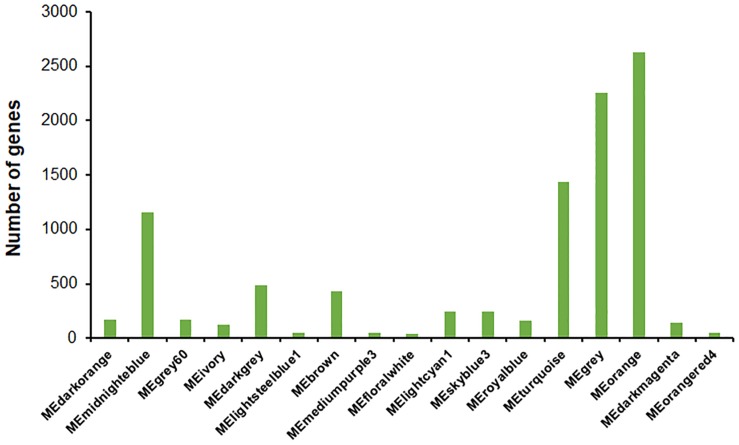
Gene numbers in each module. Number of genes contained in each module were generated using the Dynamic Tree Cut method and setting module dissimilarity coefficients of ≤ 0.25 as a threshold for module merging.

A total of 45 modules were identified using the Dynamic Tree Cut method (Langfelder and Horvath, 2007, 2008; Figure 7D). Further merging of modules generated 17 modules with set module dissimilarity coefficients of ≤ 0.25 (Figure 7D). Members of these 17 modules showed variances, ranging from 36 to 2,623 (Figure 8). Of these, MEgrey contained genes that were not co-expressed. The genome of *P*. *oxalicum* contained 35 genes annotated as cellulases and *XYNs* (Zhao et al., 2016) that were further analyzed. They were divided into seven modules, MEivory, MEmidnightblue, MEorange, MEroyalblue, MEturquoise, MEgrey and MElightcyan1. WGCNA analysis of *T. reesei* grown on sugarcane bagasse identified 28 highly connected gene modules and one of these modules contained the most representative core of cellulolytic enzymes, with their regulators and transporters (Borin et al., 2018).

Module MEivory contained 120 members (Supplementary Table S4), and their encoded proteins included 33 CAZymes, nine putative TFs, 15 transmembrane proteins and several proteins involved in protein homeostasis. Of these, 24 of the 33 CAZymes were annotated as plant cell wall-degrading enzymes (CWDEs), i.e., the three CBHs POX05587/Cel7A-2, POX04786/Cel6A and POX02490/Cel7A-1, seven EGs POX05570/Cel45A, POX01896/Cel5C, POX01166/Cel5B, POX06147/Cel5A, POX05571/Cel7B, POX02740 and POX06983, four BGLs POX01437, POX03641, POX06079 and POX03062, two XYNs POX00063/Xyn10A and POX08484/Xyn11B and two polygalacturonases POX04815 and POX05580, as well as the lytic polysaccharide monooxygenase POX08897/AA9, expansin-like POX08485, α-galactosidase POX02345, β-1,4-mannanase and β-xylosidase POX01646. Previous secretomic observations of *P*. *oxalicum* revealed that most of these CWDEs were secreted into the extracellular space (Zhao et al., 2016), suggesting that MEivory contains most of the secreted cellulases and xylanases produced by *P*. *oxalicum*.

Among the nine putative TF genes, four genes, *POX01960/ClrB*, *POX02071/ClrB-2*, *POX03837/ClrA* and *POX05324/XlnR*, encoded Zn2Cys6 proteins that are involved in the regulation of cellulase and/or xylanase production in filamentous fungi (Coradetti et al., 2012; Li et al., 2015; Zhao et al., 2016). ClrB up-regulates cellulase and xylanase production, except for pNPGase (Zhao et al., 2016), while a deletion of *ClrB-2* partially reduces the cellulase activity of *P. oxalicum* (Li et al., 2015). XlnR down-regulates cellulase production while up-regulating xylanase production (Li et al., 2015). Both ClrB and XlnR dynamically control the transcriptional levels of major cellulase and/or xylanase genes under the induction of cellulose, but their precise roles differ depending on the species concerned (Benocci et al., 2017). ClrA/Clr-1 is not only required for the expression of major cellulase and xylanase-encoding genes, but is also necessary for the expression of *ClrB*/*Clr-2* (Coradetti et al., 2012). However, whether the five remaining TF genes *POX01118*, *POX01474*, *POX01678*, *POX04007* and *POX07871* participate in the regulation of cellulase and xylanase production of *P. oxalicum*, needs to be further studied.

Among the 15 transmembrane proteins, *POX06051*, encoding a key cellodextrin transporter POX06051/CdtC, is required for cellobiose consumption and the expression of major cellulase genes in *P. oxalicum*, in combination with another transporter, POX05915/CdtD (Li et al., 2013), suggesting the crucial role of sugar transporters in the induction of cellulase gene expression by insoluble cellulose. In addition, the inductive roles of sugar transporters exhibit redundancy (Li et al., 2013). Here, seven of the remained ten genes encoding transmembrane proteins were annotated as sugar/inositol transporters (IPR003663) and/or members of the major facilitator superfamily (IPR011701), without well-characterized biological functions. *POX00907*, *POX01263*, and *POX07722* were predicted to encode a cytochrome P450, an integral membrane protein and a bicarbonate transporter, respectively. Two candidate classic G protein-coupled receptors POX01263 and POX03930 containing seven transmembrane domains (TMDs) had a specific feature of classic GPCRs (Martín et al., 2019). However, POX03930 was annotated as a RAT1-like protein that is involved in fungal detoxification (Soustre et al., 1996).

The remaining 63 genes of 120 members in module MEivory included three genes (*POX05586*, *POX01637*, and *POX07741*) involved in metabolism, three genes (*POX03367*, *POX07086*, and *POX07745*) involved in genetic information processing, one gene (*POX01689*) involved in environmental information processing, and 56 unknown genes, through the annotation using KEGG database. *POX03367* encoding putative ATP-dependent Clp protease POX03367/ClpX, and *POX07086* and *POX07745* encoding disulfide-isomerases were reported to be associated with protein homeostasis (Freedman et al., 2017; Bell et al., 2018). Moreover, protein POX01689 shared 62.0% identity with Ras GTase Rsr1 (accession number CAA59809.1), which is involved in the regulation of cell polarity in *Saccharomyces cerevisiae* (Miller et al., 2019). POX05586, POX01637, and POX07741 were annotated as formyltetrahydrofolate deformylase PurU, catechol *O*-methyltransferase COMT and DNA polymerase epsilon subunit 2 POLE2, respectively.

The secondary module MEroyalblue contained 157 members (Supplementary Table S5), including seven cellulase and XYN genes (a *bgl POX08882*, an *eg POX07535/Cel12A* and five *xyns POX04274, POX05916, POX06601, POX06783/Xyn11A* and *POX08990*) and 22 other CWDE genes. Notably, most of the xylanolytic genes in the HP7-1 genome were included in MEroyalblue, such as five *xyns* described above and eight β-xylosidase genes (*POX00007, POX01914, POX01921, POX05540, POX06599, POX06600, POX07441*, and *POX07891*). Only two TF genes *POX03888/PrtT* and *POX06865* were included. POX03888/PrtT, a transcription activator of extracellular proteases in filamentous fungi, could directly regulate the expression of many peptidase genes and several amylase genes including α-amylase, glucoamylase and α-glucosidase genes. POX03888/PrtT also indirectly regulated the expression of cellulase genes, which may be related to nutrient limitations (Chen et al., 2014). However, to our knowledge, there is no report about its regulation of xylanolytic genes in *P. oxalicum*. MEroyalblue also contained 19 transmembrane proteins with 2–14 TMDs, most of which were annotated as sugar/inositol transporter (IPR003663) and/or major facilitator superfamily (IPR011701), including cellodextrin transporter POX05915/CdtD.

The remaining 92 of 157 members in module MEroyalblue included 38 genes involved in metabolism (i.e., amino acid metabolism, carbohydrate metabolism, energy metabolism, lipid metabolism and enzyme families) and 54 unknown genes through KEGG annotation. Notably, four genes *POX02832*, *POX02775*, *POX05829*, and *POX07164*, encoded putative aldose 1-epimerase GalM, alcohol dehydrogenase, alcohol dehydrogenase and triosephosphate isomerase TpiA, respectively, which participate glycolysis/gluconeogenesis. However, these annotated genes were not previously known to be associated with cellulase and/or xylanase production in *P. oxalicum*.

The remaining five modules MEmidnightblue, MEorange, MEturquoise, MEgrey, and MElightcyan1 contained a few unimportant cellulase and XYN genes, and their members accounted for approximately 80% of all genes annotated in the genome of HP7-1 (Zhao et al., 2016), which should not therefore, be co-expressed with the major cellulase and XYN genes.

**FIGURE 9:**
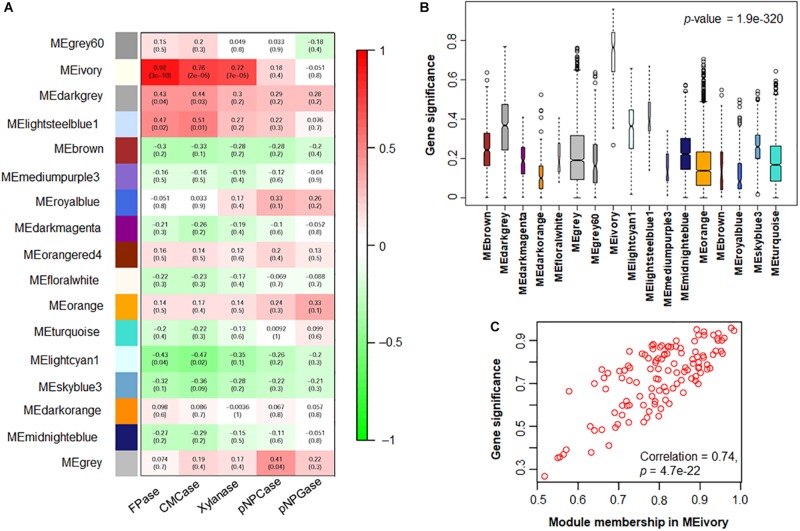
Module and trait correlations. **(A)** Modules and cellulase and xylanase production associations. The rows and columns correspond to module eigengenes and enzyme production levels, respectively. Each cell indicates the correlation coefficient and *p*-value between the module and specific enzyme production level. **(B)** Heatmap plot of mean gene significances across modules. **(C)** Gene significance vs. module membership in MEivory, which correlated the most with traits in panel **(A)**.

In order to understand the significance of the identified modules toward cellulase and xylanase production of *P. oxalicum*, their correlation coefficients were calculated. Using correlation coefficient > 0.4 and *p*-value < 0.05 as thresholds, the relevant modules to cellulase and xylanase production of *P. oxalicum* were screened and characterized. The correlation strengthens with the increase in the correlation coefficient. Module MEivory was most relevant to filter-paper cellulase and CMCase, with correlation coefficients of 0.92 (*p*-value 3e−10) and 0.76 (*p*-value 2e−5), respectively, followed by MEdarkgrey (0.43, *p*-value 0.04, and 0.44, *p*-value 0.03, respectively) and MElightsteelblue1 (0.47, *p*-value 0.02, and 0.51, *p*-value 0.01, respectively). MElightcyan1 showed low negative correlations (−0.43, *p*-value 0.04 and −0.47, *p*-value 0.02) with FPase and CMCase production, respectively, whereas no module was found to be correlated with pNPCase or pNPGase production. Only MEivory correlated with xylanase production of *P. oxalicum* (0.72, *p*-value 3e−10; Figure 9A). These results were confirmed through gene and module significance analyses (Langfelder and Horvath, 2008; Figures 9B,C).

**FIGURE 10:**
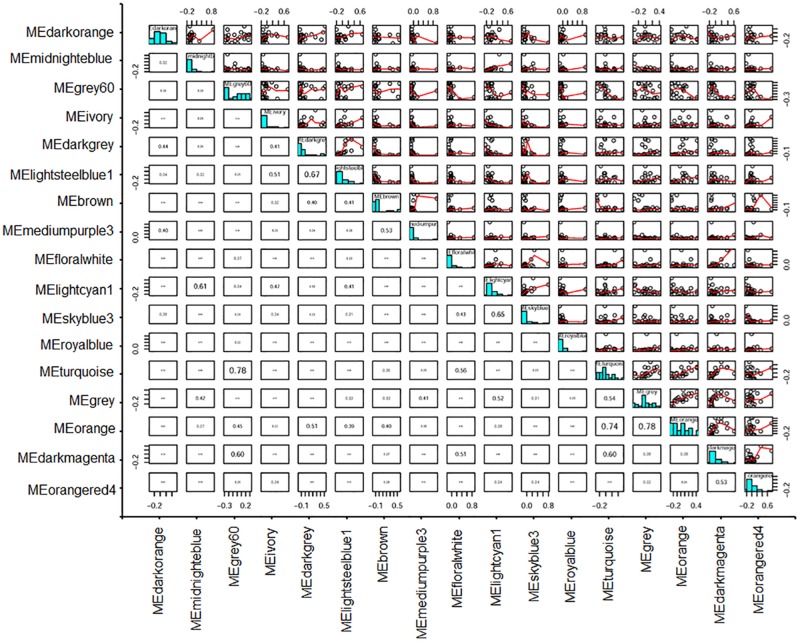
Heatmap plot of correlations between module eigengenes. Values in each cell represent the correlation coefficients between the adjacent modules.

In addition, a relationship assay between module eigengenes indicated that MEivory had relatively tight correlations with MElightsteelblue1, MElightcyan1, and MEdarkgrey, compared with other modules, having correlation coefficients of 0.51, 0.47, and 0.41 (Figure 10).

The modules, MEdarkgrey, MElightcyan1 and MElightsteelblue1, included 490, 240, and 46 members, respectively. Surprisingly, several genes encoding proteins involved in fungal conidiation were observed in MEdarkgrey, including POX00872/NimX, POX01176/ArpA-like, POX01430/Alb1/wA, POX03412/ArpA, POX05090/TmpA, POX06534/BrlA, POX07586/FlbB, POX07025/AbaA, POX07099/FlbD, POX01179/ArpB-like, POX01390/AygA and POX01391/AbrA, indicating that MEdarkgrey might be a co-expression network involved in fungal conidiation. Fungal conidiation is associated with several environmental factors such as nutrient limitation and light (Ruger-Herreros and Corrochano, 2020). For example, GLU starvation induces hydrolase production and maturation, while GLU richness enhances mycelial biomass and conidiation. The secretion of extracellular hydrolases supports conidia formation by releasing nutrients (Emri et al., 2018) such as GLU, resulting in the co-regulation of fungal conidiation and hydrolase production. Two key members of the central regulatory pathway, BrlA and AbaA, tightly control fungal sporulation, acting in concert with fluffy low *BrlA*s (FLBs), such as FlbB and FlbD (Park and Yu, 2012). BrlA down-regulated cellulase activity of *P. oxalicum* (Qin et al., 2013), while AbaA acted positively (data not shown). Proteins AygA, AbrA, Alb1/wA, ArpA, and ArpB are involved in pigment biosynthesis (Mayorga and Timberlake, 1990; Park and Yu, 2012), and NimX, acyclin-dependent kinase, controls fungal cell division (McGuire et al., 2000). TmpA is a putative membrane flavoprotein that regulates asexual development in *Aspergillus nidulans* (Soid-Raggi et al., 2006). Whether these proteins are involved in cellulase and xylanase production of *P. oxalicum* needs to be studied further.

In MElightcyan1, the gene *POX07254* encodes an essential TF, CreA, that mediates fungal carbon catabolite repression in the presence of the favored carbon source, GLU. CreA inhibits the expression of almost all the cellulase and xylanase-encoding genes and their regulatory genes, including *ClrB* and *XlnR*, induced by insoluble cellulose (Antonieto et al., 2014; Li et al., 2015; Huberman et al., 2016). In contrast, the other genes in this module have neither been found to be associated with the regulation of cellulase and xylanase-encoding genes in fungi, nor as genes in module MElightsteelblue1.

### Identification of Highly Connected (Hub) Genes in MEivory

**FIGURE 11:**
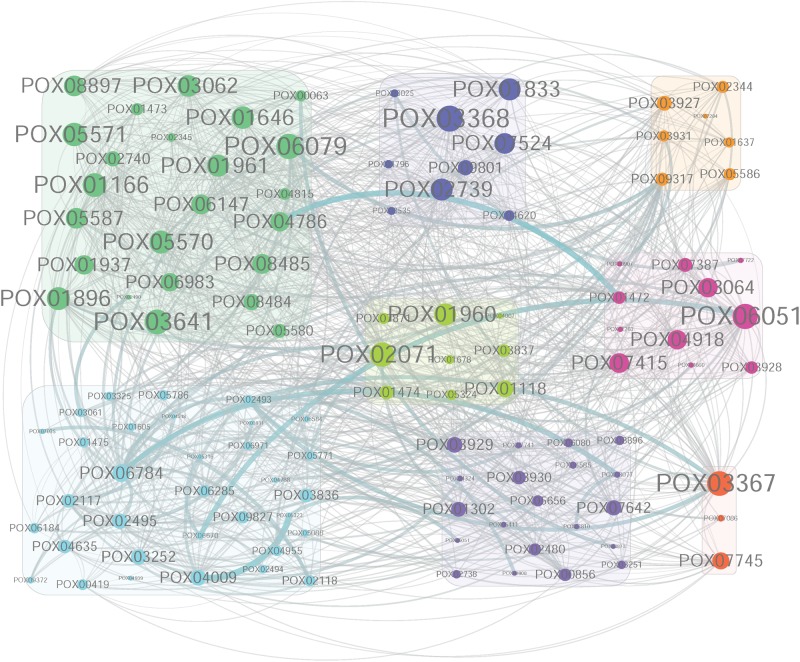
Co-expression network of the highly connected genes in module MEivory. The co-expression network of genes in MEivory was constructed using Cytoscape 3.6.1 software. This network only shows the genes having correlation coefficients greater than 0.4. Cycle size indicates the number of connected genes. Green, CWDEs; Blue, the remaining CAZymes; Light-green, TFs; Dark-orange, proteins involved in protein homeostasis; Orange, other annotated proteins; Pink, putative transporters; Light-blue, hypothetical proteins; Purple, transferases.

The co-expression network of genes in MEivory was constructed using Cytoscape 3.6.1 software (Shannon et al., 2003), and the hub genes were investigated in the constructed network. The 20 largest hub genes in MEivory were *POX03368*, *POX06079*, *POX06051/CdtC*, *POX03367*, *POX03641*, *POX02071*, *POX01166/Cel5B*, *POX05571/Cel7B*, *POX05570/Cel45A*, *POX01896/Cel5C*, *POX01646*, *POX02739*, *POX01961*, *POX01960*/*ClrB*, *POX01833*, *POX03062*, *POX05587*/*Cel7A-2*, *POX08897*/*AA9*, *POX07524*, and *POX07415*, having 34 to 48 coupled genes (Figure 11). Most of them encoded proteins that were CAZymes, including the essential cellulases (CBH POX05587/Cel7A-2; EGs POX01166/Cel5B, POX05571/Cel7B, POX05570/Cel45A and POX01896/Cel5C; BGLs POX01646, POX03062, POX06079, and POX03641; Auxiliary activity POX08897/AA9), and their regulators such as *POX01960*/*ClrB* and *POX02071*/*ClrB-2*. It is not surprising that these cellulase genes and their regulatory genes in filamentous fungi including *P. oxalicum*, *T. reesei*, and *Neurospora crassa*, are clustered together because they are co-induced in response to most plant biomass substrates such as WB, sugarcane bagasse and Avicel (Craig et al., 2015; Yan et al., 2017; Borin et al., 2018). Under fungal growth conditions, in the presence of cellulose, cellulose is first digested into cellodextrins by constitutive levels of extracellular CBHs and EGs. The generated cellodextrins are transported into the fungal cells by the cellodextrin transporter CdtC, then either degraded into GLU by intracellular β-glucosidases, such as POX06079, or act as an inducer of cellulase and xylanase gene expression (Li et al., 2015). The proteins POX01646, POX01833, POX01961, POX02739 and POX07524, were annotated as β-xylosidase, α-mannosyltransferase, β-mannosidase, carbohydrate acetylesterase and α-L-rhamnosidase, respectively, and may assist the major cellulases with degradation of plant cell walls. POX03368 shared a 33.72% identity with MreA from *Aspergillus oryzae* (accession number BAB13480.1) that encodes an isoamyl alcohol oxidase. Isoamyl alcohol oxidase catalyzes the formation of isovaleraldehyde as the main component of *mureka*, producing the off-flavor (Yamashita et al., 2000). POX03367 was annotated as an ATP-dependent Clp protease, ATP-binding subunit ClpX. ClpX is a protein chaperone of ClpP proteases, which are responsible for protein homeostasis through the degradation of damaged or unfolded proteins, as well as for the conditional degradation of functional proteins in response to environmental signals (Stahlhut et al., 2017). The function of ClpXP has been studied broadly in prokaryotes but rarely reported in fungi. Whether POX03367 and POX03368 are involved in the regulation of cellulase and xylanase-encoding gene expression levels in fungi remains unknown, and needs to be further studied.

### Kinetic Analysis of the Expression Levels of MEivory Members

**FIGURE 12:**
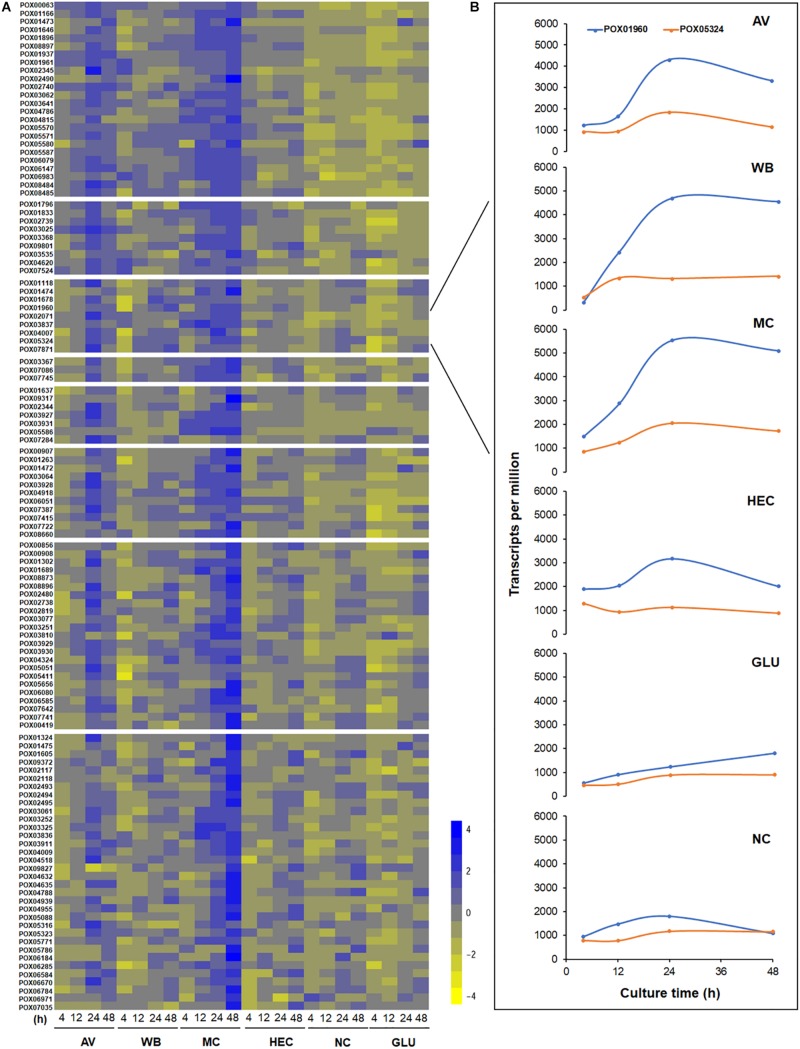
Heatmap plot of gene expression levels in module MEivory **(A)** and a comparison of transcription levels between *POX01960* and *POX05324* **(B)**. The heatmap **(A)** is constructed based on log2 fold change of each gene on different carbon sources compared with that on NC; transcript plots **(B)** use the FPKM of each gene. AV, Avicel; WB, wheat bran; MC, methyl cellulose; HEC, 2-hydroxyethyl cellulose; GLU, glucose; NC, no carbon source; FPKM, fragments per kilobase of exon per million mapped reads.

**FIGURE 13:**
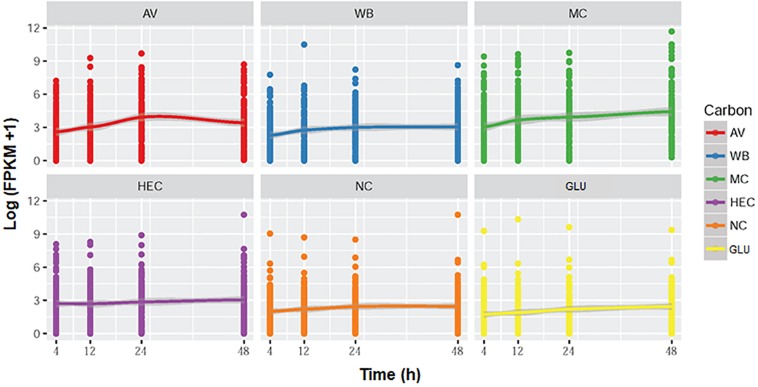
Graphs showing the expression of genes (FPKM) in module MEivory over time. AV, Avicel; WB, wheat bran; MC, methyl cellulose; HEC, 2-hydroxyethyl cellulose; GLU, glucose; NC, no carbon source; FPKM, fragments per kilobase of exon per million mapped reads.

The overall expression levels of these 120 genes were induced by complex carbon sources (i.e., AV, MC, WB, and HEC), but the levels varied depending on the carbon source. Conversely, their transcriptional levels in both the presence of GLU and NC were low or inhibited (Figure 12A). Comparative analyses revealed that the expression levels of MEivory members increased between 4 and 24 h of induction by AV and then decreased, while remaining increased over time by MC. In contrast, their expression only increased from 4 to 12 h and then stabilized in the presence of HEC or WB. The expressional levels of MEivory in the presence of MC and AV were higher than those on HEC and WB, especially at later stages of culture (Figure 13). The overall expression changes over time should be attributed to the distinct structure and chemical composition of different biomass substrates (Wang et al., 2015). AV has a high crystallinity, in which large macrofibrils are aggregated together, resulting in a tightly packed structure, which limits the penetration of cellulolytic enzymes. Therefore, degradation of AV is based on a layer-by-layer model, with synergistic interactions between CBHs, EGs and BGLs (Yang et al., 2011; Bornscheuer et al., 2014). During the early stage of AV induction, *P. oxalicum* stimulates the expression of genes in MEivory to degrade AV and promote GLU uptake, which increases over time. However, during the later stages, the secreted cellulolytic enzymes were enough for the degradation of the remaining substrate, or fungal cells might have required little GLU as a result of maturation or death, which contributed to the reduction in gene expression. MC and HEC are derived from cellulose, through random methylation and hydroxyethylation, respectively. Theoretically, their digestion also requires synergistic action by several cellulolytic enzymes. However, the methylated and hydroxyethylated groups should inhibit the binding of enzymes to these substrates, which might lead to a different enzyme composition. In addition, methylated and hydroxyethylated cello- or mono-oligosaccharides might enter into fungal cells to differentially induce gene expression, but this needs confirmation. In contrast, WB is composed predominantly of starch, non-starch polysaccharides such as cello-oligosaccharides and GLU, and crude proteins (Sardari et al., 2019). Cello-oligosaccharides are required for cellulase and xylanase production in *P. oxalicum* (Sun et al., 2008). However, how WB induces the expression of genes encoding cellulolytic enzymes has been unclear up to now. GLU is known to repress the secretion of cellulolytic enzymes in filamentous fungi through promoting carbon catabolite repression (CCR). In the presence of GLU, CCR is regulated mainly by transcription factor CreA, that represses the expression of genes encoding plant cell wall-degrading enzymes and their regulatory genes. The regulation of CreA partially depends on its intracellular localization and phosphorylation (Huberman et al., 2016). Under growth induction conditions, CreA forms a CreA-SsnF-RcoA repressor complex that may promote CreA degradation, connected to an SCF ubiquitin ligase complex, via GskA protein kinase, whereas, when supplied with GLU, CreA disconnects from the ubiquitin ligase complex and is then transported into the nucleus of *A. nidulans* (de Assis et al., 2018).

The transcripts of two key TF genes, *POX01960/ClrB* and *POX05324/XlnR*, directly regulate the expression of major cellulase and xylanase-encoding genes in *P. oxalicum*. Transcription of *POX01960/ClrB* was higher than that of *POX05324/XlnR* during the whole culture time on each carbon source (Figure 12B), suggesting that POX01960/ClrB plays a much greater role in the regulation of cellulase and xylanase-encoding gene expression in *P*. *oxalicum* than POX05324/XlnR. This finding is in contrast to Xyr1 and XlnR in *T. reesei* (Mach-Aigner et al., 2008; Borin et al., 2018), which was confirmed through the previous genetic analysis (Li et al., 2015; Zhao et al., 2016).

### Differential Expression Levels of MEivory Members Induced by Various Carbon Sources

Among the 120 members of MEivory, 22–108 DEGs were detected in the presence of five carbon sources (AV, MC, HEC, WB, and GLU; Supplementary Figure S12). Comparative analyses indicated that all four complex carbon sources (but not GLU) could co-activate the expression of 12 DEGs over the whole culture time and their encoding proteins included nine CAZymes (four EGs, POX02740, POX05570/Cel45A, POX05571/Cel7B and POX06147/Cel5A, one BGL, POX06079, one XYN, POX00063/Xyn11A, a β-1,4-mannanase, POX01937, a β-mannosidase, POX01961 and an α-mannosyltransferase, POX01833), the cellodextrin transporter POX06051/CdtC and two unknown proteins POX03929 and POX03930 (Supplementary Table S4). The InterPro (IPR) annotation revealed that POX03929 and POX03930 were an RTA-like protein (IPR007568), with multiple transmembrane regions, and a P-loop, containing nucleoside triphosphate hydrolase (IPR027417), respectively. To facilitate further study, these 12 DEGs were named as basic co-expression genes under biomass induction.

In addition, all 12 DEGs increased their transcriptional levels to various degrees under the induction of AV, MC, HEC and WB, with log2 fold changes from 1.1 to 10.3, except *POX01833* on WB at 4 h. Conversely, most of them showed a significant reduction on GLU (Figure 12A).

**FIGURE 14:**
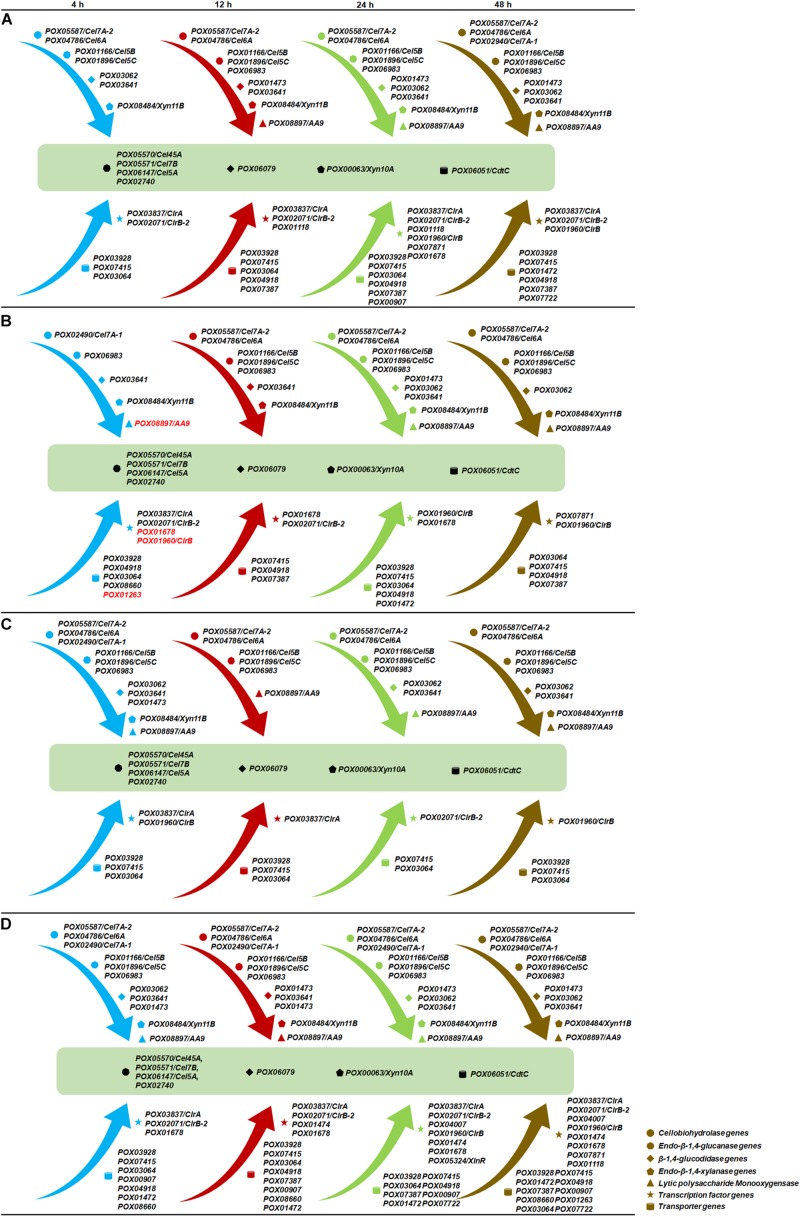
Model of sequential gene expression in response to various carbon sources in *P. oxalicum*. **(A)** Avicel; **(B)** wheat bran; **(C)** 2-hydroxyethyl cellulose; **(D)** methyl cellulose. Gene IDs in black indicate up-regulated genes on the corresponding carbon sources compared with no carbon source, while gene IDs in red indicate down-regulated genes. Genes in dark green were co-activated by all four induced carbon sources over the whole culture time. Arrows in blue, red, green and brown represent different induction times, recoding as 4, 12, 24, and 48 h, respectively.

In addition to these 12 DEGs that were induced commonly by biomass, the expression levels of several specific DEGs showed significant alterations that were dependent on the duration of induction and/or different substrates. For example, the expression levels of two key *cbh* genes, *POX05587/Cel7A-2* and *POX04786/Cel6A*, were activated at 4 h on AV, HEC and MC, but not on WB, compared with NC. The regulatory gene *POX01960*/*ClrB* was induced after 24 h on AV and MC, as well as at 4 and 48 h on HEC, but was inhibited at 4 h on WB. The transcription levels of *POX05324/XlnR* on all five carbon sources showed no significant differences from NC, except at 24 h on MC (Figures 12, 14).

Consequently, the expression levels of major cellulase and xylanase genes in *P. oxalicum* were simultaneously induced, but at different expression levels, thereby resulting in different enzyme production levels. The expression changes may be attributed to different sites of action of the different cellulolytic enzymes and the distinct chemical composition of different biomass substrates (Wang et al., 2015). EGs randomly attack internal β-1,4-glycosidic bonds in cellulose chains to generate numbers of cello-oligosaccharides with exposed ends. CBHs release cellobiose from both ends of cello-oligosaccharides. Finally, BGLs break cellobiose and cello-oligosaccharides into GLU (Wang et al., 2013). Atomic force microcopy indicated that the rough surface of the original AV became smoother after enzymatic hydrolysis, which results from the expression of the essential *cbhs* and *egs* during the early induction period (Yang et al., 2011; Bornscheuer et al., 2014); the AV derivatives HEC and MC behaved similarly. In contrast, WB is composed predominantly of starch, non-starch polysaccharides and crude proteins. The non-starch polysaccharides included cello-oligosaccharides, arabinoxylans and β-(1,3) (1,4)-glucan (Sun et al., 2008). Therefore, when cultivated on WB, *P. oxalicum* could use GLU from the degradation of other polysaccharides, such as starch, during the early induction period.

Altogether, comparative transcriptomic analysis revealed that the transcriptional levels of 964 DEGs encoding putative CAZymes, TFs, transporters and GPCRs, namely common DEGs (Supplementary Table S6), showed alterations at various degrees in response to the chosen carbon sources AV, WB, MC, HEC, and GLU, compared with that on NC. Among them, at 4 h of induction, a total of 15 CAZyme-encoding genes, 11 transporter-encoding genes and 13 TF-encoding genes simultaneously altered their transcriptional levels in the presence of all the carbon sources, whereas no GPCR-encoding genes did. There were two (i.e., only TF-encoding genes), 176 (i.e., 66 CAZyme-encoding genes, 42 transporter-encoding genes, 56 TF-encoding genes, and 12 GPCR-encoding genes), ten (i.e., six CAZyme-encoding genes, three transporter-encoding genes and one TF-encoding genes), nine (i.e., four CAZyme-encoding genes, one transporter-encoding genes and four TF-encoding genes) and 97 DEGs (i.e., 46 CAZyme-encoding genes, 31 transporter-encoding genes and 20 TF-encoding genes) showing specific to AV, WB. MC, HEC, and GLU, respectively. At 12–48 h, 6–10 CAZyme-encoding genes, 6–10 transporter-encoding genes, and 5–7 transporter-encoding genes showed response to all carbon sources. A total of 13–44, 49–63, 25–79,1–8, and 61–175 common DEGs were specific to AV, WB. MC, HEC, and GLU, respectively (Supplementary Table S7).

### Identification of the Uncharacterized TFs for Regulating Fungal Cellulase and Xylanase Activities

**FIGURE 15:**
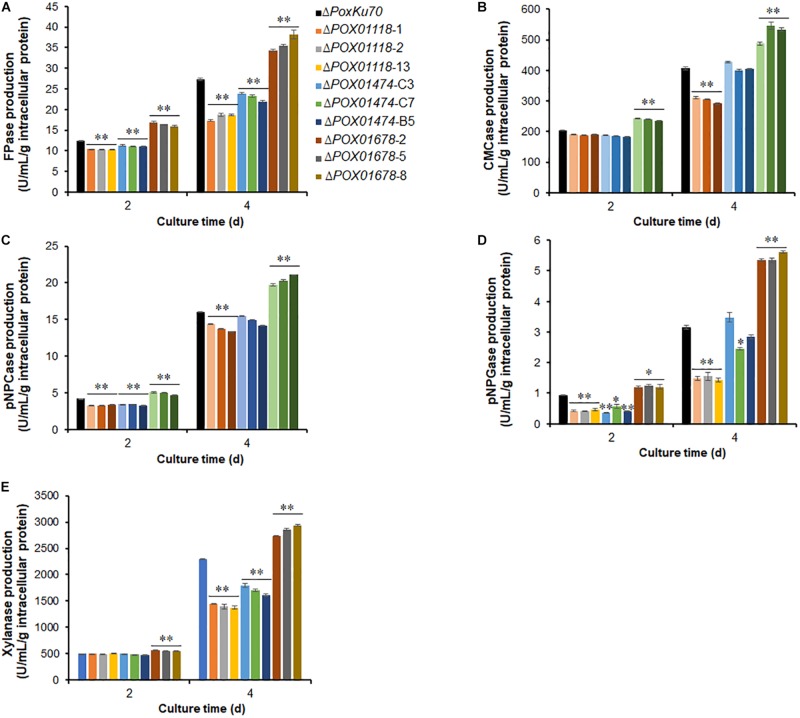
Cellulase and xylanase activities of three *P. oxalicum* deletion mutants Δ*POX01118*, Δ*POX01474*, Δ*POX01678* and the parental strain Δ*PoxKu70*, cultivated in medium containing Avicel after a transfer from GLU. **(A)** FPase; **(B)** CMCase; **(C)** pNPCase; **(D)** pNPGase; **(E)** xylanase. The experiments were carried out using three independent transformants of each gene. Enzymatic activities were determined at 2–4 days of cultivation. Enzymes activities were normalized by total intracellular proteins. ^∗^*p* ≤ 0.05 and ^∗∗^*p* ≤ 0.01 indicate significant differences between deletion mutants and parental strain Δ*PoxKu70* by Student’s *t-*test. FPase, filter paper cellulase; CMCase, carboxymethylcellulase; pNPCase, *p*-nitrophenyl-β-cellobiosidase; pNPGase, *p*-nitrophenyl-β-glucopyranosidase.

Weighted gene co-expression network analysis revealed that the expression of nine TF genes was co-expressed with major cellulase and xylanase-encoding genes in *P. oxalicum*, of which four were known. We further constructed the deletion mutants of three genes, *POX01118*, *POX01474*, *POX01678* (Supplementary Figure S13) and investigated their regulatory roles in cellulase and xylanase production. Unfortunately, deletion of *POX04007* and *POX07871* that encoded C2H2- and Zn2Cys6-zinc finger proteins failed. Enzymatic activity assays indicated that mutants Δ*POX01118* and Δ*POX01474* lost approximately 30% of their cellulase and xylanase activities under some induction conditions, compared with Δ*PoxKu70*. For example, Δ*POX01118* had decreased FPase activity by 31.6–36.8% after 4 day of Avicel induction, while Δ*POX01474* had decreased xylanase activity by 22.0–29.8% at 4 day. Conversely, Δ*POX01678* had increased cellulase and xylanase activities (by approximately 30%) corresponding to FPase, CMCase, pNPCase and pNPGase, as well as xylanase activity (*p* < 0.05; Figure 15).

Simple Modular Architecture Research Tool (SMART) analysis^5^ indicated that POX01118 contained a GAL4-like Zn2Cys6 binuclear cluster DNA-binding domain (IPR001138; *E*-value 0.00000175) at the *N*-terminus (Supplementary Figure S14a). POX01118 shared 99.9% and 63.9% identity with PDE_09179 (GenBank accession number EPS34215.1) in *P. oxalicum* strain 114-2 and ANI_1_346064 (XP_001391371.2) in *Aspergillus niger* strain CBS 513.88, respectively. Phylogenetic analysis indicated that POX01118 was close to the homologs in *Aspergillus* and *Talaromyces* (Supplementary Figure S14b).

POX01474 contained a GAL-like domain (IPR001138; *E*-value 6.6e−7), a fungal trans domain (IPR007219; *E*-value 0.004; Supplementary Figure S14a), and shared 99.7% and 52.7% identity with PDE_09536 (EPS34572.1) in *P. oxalicum* strain 114-2 and citrinin biosynthesis transcriptional activator ctnR (TVY74105.1) in *Fusarium oxysporum* f. sp. *cubense* strain 160527, respectively. POX01474 is conserved in *Penicillium* and some members of *Aspergillus* (Supplementary Figure S14c).

POX01678 contained three ‘SANT, SWI3, ADA2, N-CoR, and TFIIIB’ (SANT) DNA-binding domains (IPR001005; *E*-values 0.00905, 4.38e−10, and 8.98e−7; Supplementary Figure S14a), and shared 97.5% and 45.4% identities with PDE_09744 (EPS34780.1) in *P. oxalicum* strain 114-2 and TCE0_017f04534 in *Talaromyces cellulolyticus* Y-94. POX01678 is specific to *P*. *oxalicum* (Supplementary Figure S14d).

## Conclusion

In this study, we identified a novel inducer (MC) for fungal cellulase and xylanase production and further comparatively analyzed the transcriptional patterns of *P. oxalicum* in response to MC, HEC, WB, AV, GLU, and NC. Moreover, through WGCNA analysis, the co-expression module MEivory that contains the cellulase and xylanase-encoding genes of *P. oxalicum* was identified. The transcriptional levels of MEivory members were dependent on the inducing carbon source and the induction time. Finally, three novel TFs POX01118, POX01474, and POX01678 were found to regulate the cellulase and xylanase production of *P. oxalicum*. These findings will facilitate understanding of the mechanisms required for the synthesis and secretion of fungal cellulases and xylanases and will provide a guide for *P. oxalicum* application in biotechnology.

## Data Availability Statement

The datasets generated for this study can be found in the GEO database with accession number GSE133258 on NCBI. The DNA sequences for POX01118, POX01474 and POX01678 have been deposited in the GenBank database (accession numbers MN529555–MN529557).

## Author Contributions

J-XF conceived, supervised this study, and revised the manuscript. SZ codesigned and co-supervised this study, and revised manuscript. C-XL carried out bioinformatic analyses, mutant construction and enzymatic activity assay, and manuscript preparation. X-ML was involved in preparation of experimental materials. All authors read and approved the final version of the manuscript.

## Conflict of Interest

The authors declare that the research was conducted in the absence of any commercial or financial relationships that could be construed as a potential conflict of interest.

## Footnotes

1 www.genome.gov/sequencingcostsdata

2 https://cran.r-project.org/web/packages/pheatmap/

3 https://cran.r-project.org/web/packages/compHclust/index.html

4 https://cran.r-project.org/web/packages/Hmisc/

5 http://smart.embl.de/
