# Supplementary material for: Weighted Gene Co-expression Network Analysis Identifies Critical Genes for the Production of Cellulase and Xylanase in Penicillium oxalicum
Source: Front Microbiol. 2020 Mar 27;11:520. doi: 10.3389/fmicb.2020.00520 (PMC7118919; doi:10.3389/fmicb.2020.00520)
Supplement: Supplementary file 1 [file Data_Sheet_1.PDF]

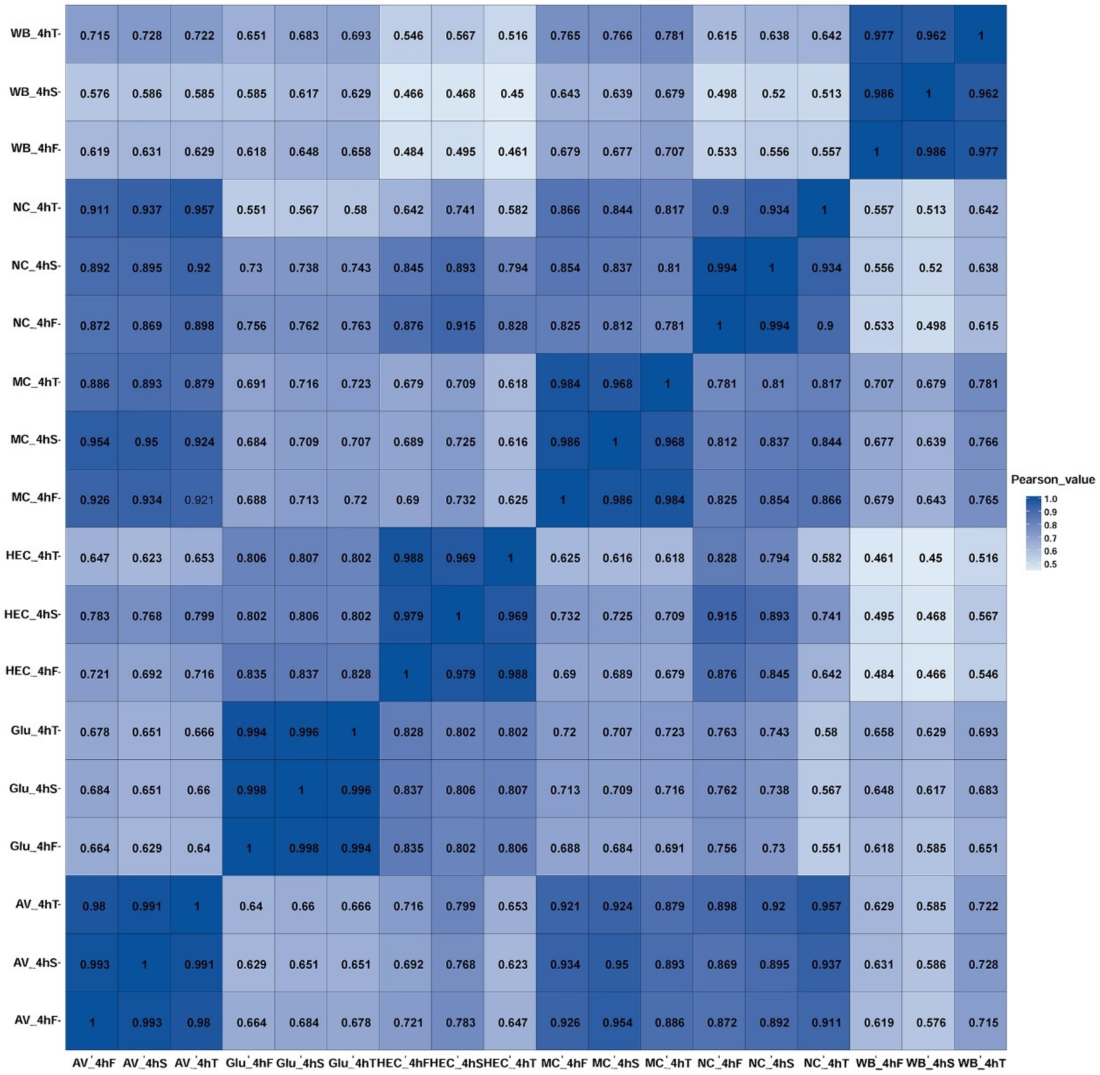

Supplementary Figure S1. Pearson's correlation analysis of the transcriptomes of *P. oxalicum* strain ∆*PoxKu70* in the presence of the five carbon sources WB, HEC, AV, MC and GLU, respectively. The NC was used for a control. RNAs for sequencing were extracted from fungal cells sampled at 4 h after a transfer from GLU cultivated for 72 h. AV: Avicel; MC: methyl cellulose; HEC: 2-hydroxyethyl cellulose; GLU: glucose; WB: wheat bran; NC: without carbon source. F: first; S: second; T: third.

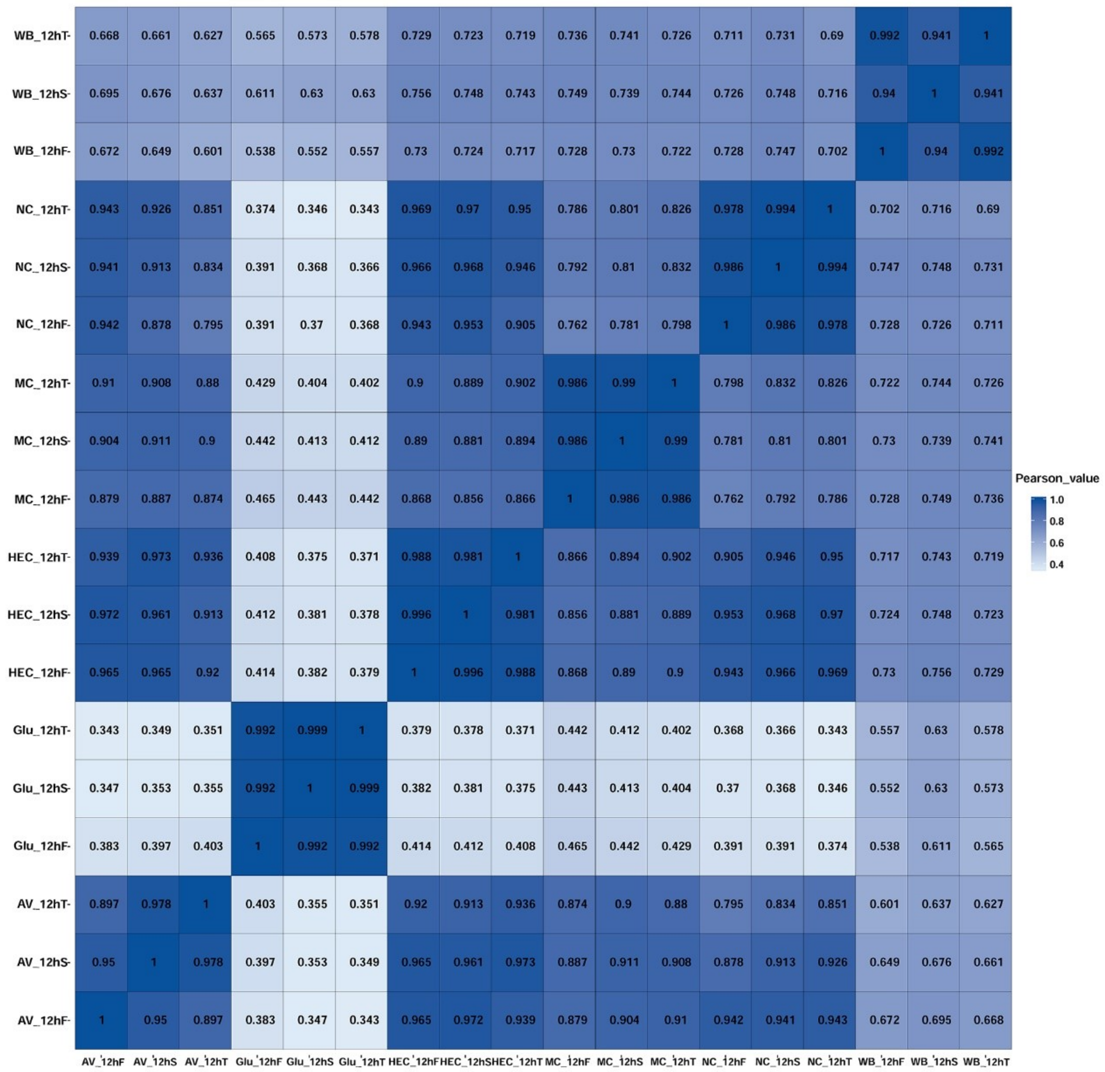

Supplementary Figure S2. Pearson's correlation analysis of the transcriptomes of *P. oxalicum* strain Δ*PoxKu70* in the presence of the five carbon sources WB, HEC, AV, MC and GLU, respectively. The NC was used for a control. RNAs for sequencing were extracted from fungal cells sampled at 12 h after a transfer from GLU cultivated for 72 h. AV: Avicel; MC: methyl cellulose; HEC: 2-hydroxyethyl cellulose; GLU: glucose; WB: wheat bran; NC: without carbon source. F: first; S: second; T: third.

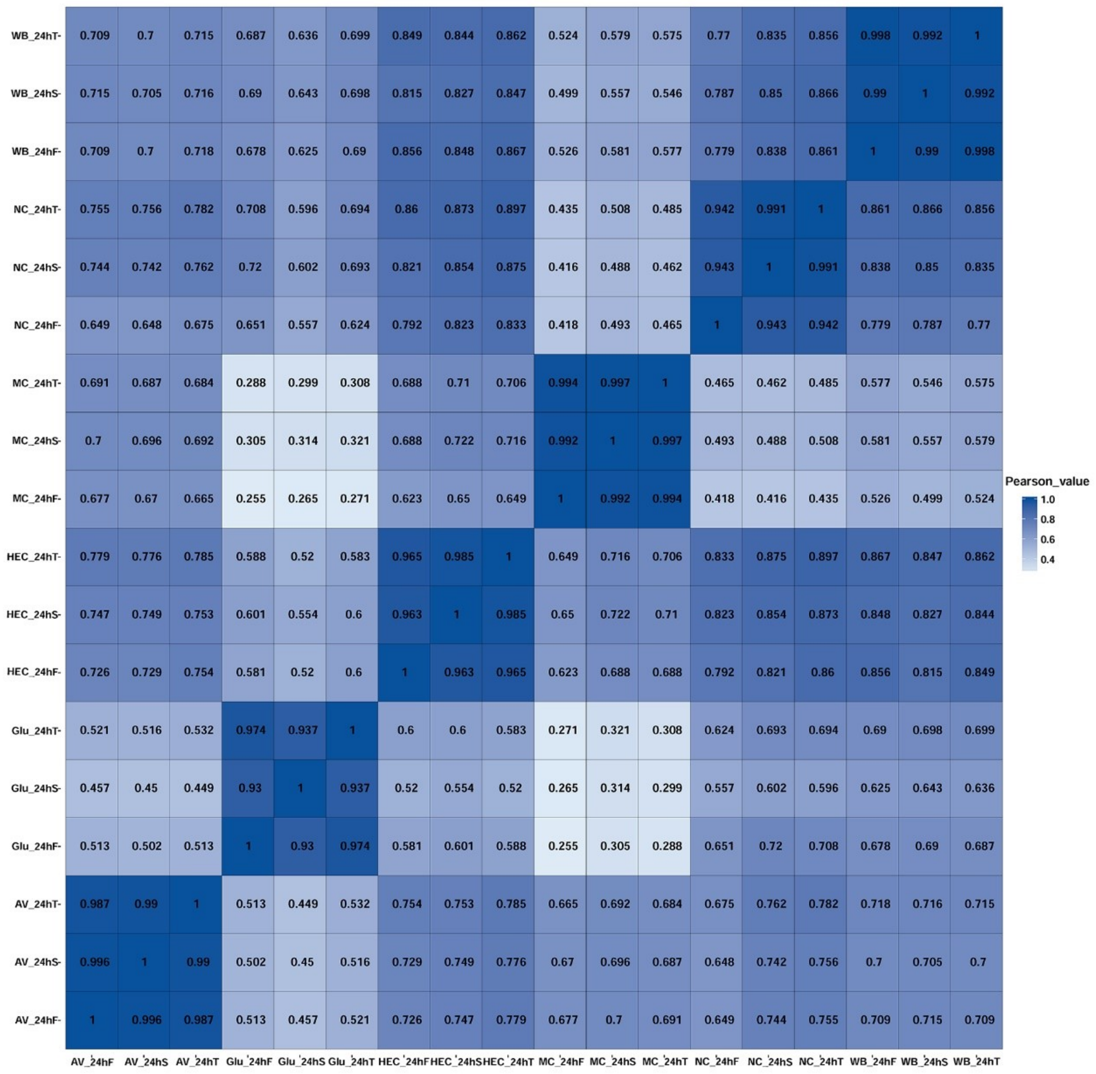

Supplementary Figure S3. Pearson's correlation analysis of the transcriptomes of *P. oxalicum* strain Δ*PoxKu70* in the presence of the five carbon sources WB, HEC, AV, MC and GLU, respectively. The NC was used for a control. RNAs for sequencing were extracted from fungal cells sampled at 24 h after a transfer from GLU cultivated for 72 h. AV: Avicel; MC: methyl cellulose; HEC: 2-hydroxyethyl cellulose; GLU: glucose; WB: wheat bran; NC: without carbon source. F: first; S: second; T: third.

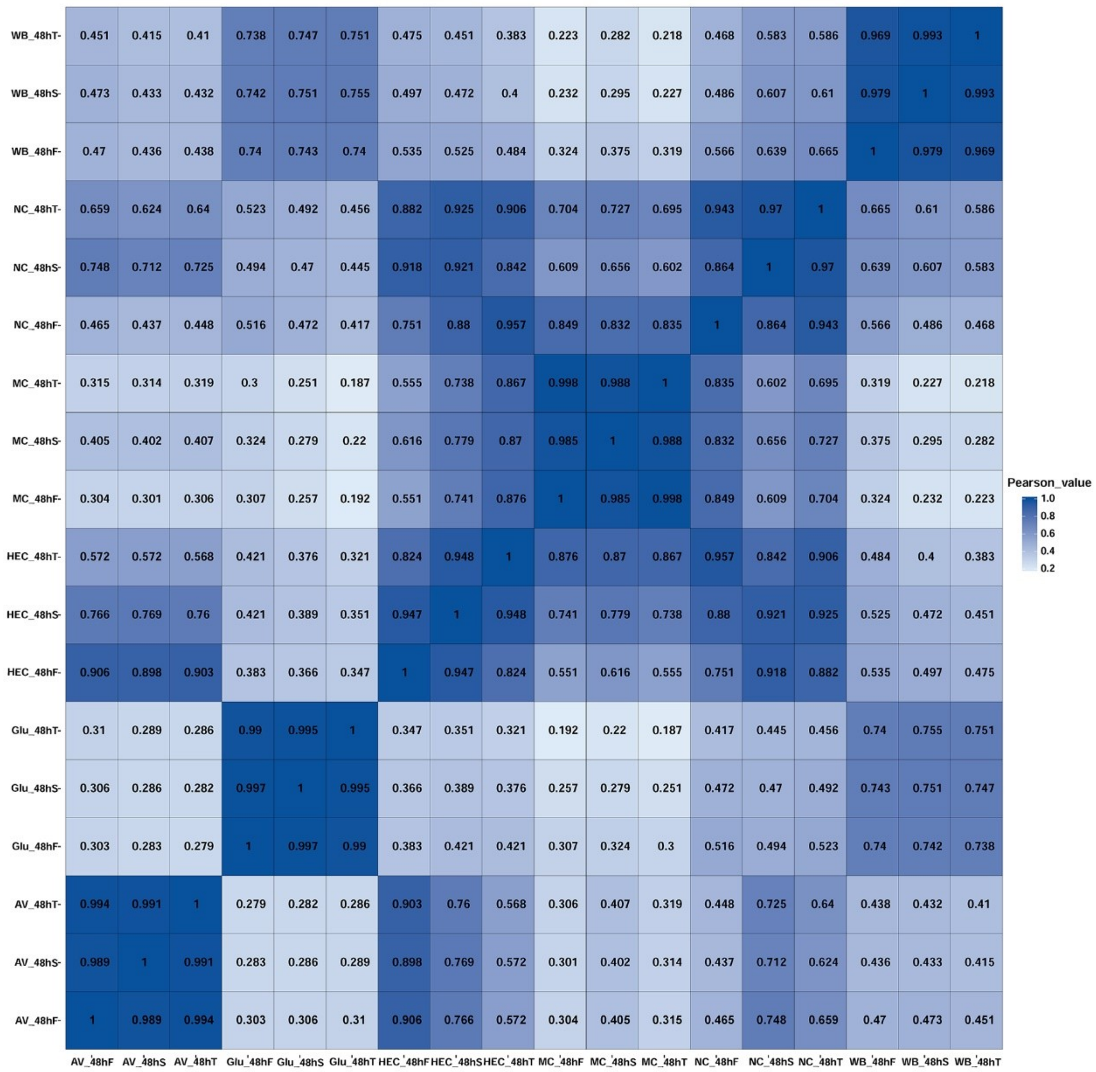

Supplementary Figure S4. Pearson's correlation analysis of the transcriptomes of *P. oxalicum* strain ∆*PoxKu70* in the presence of the five carbon sources WB, HEC, AV, MC and GLU, respectively. The NC was used for a control. RNAs for sequencing were extracted from fungal cells sampled at 48 h after a transfer from GLU cultivated for 72 h. AV: Avicel; MC: methyl cellulose; HEC: 2-hydroxyethyl cellulose; GLU: glucose; WB: wheat bran; NC: without carbon source. F: first; S: second; T: third.

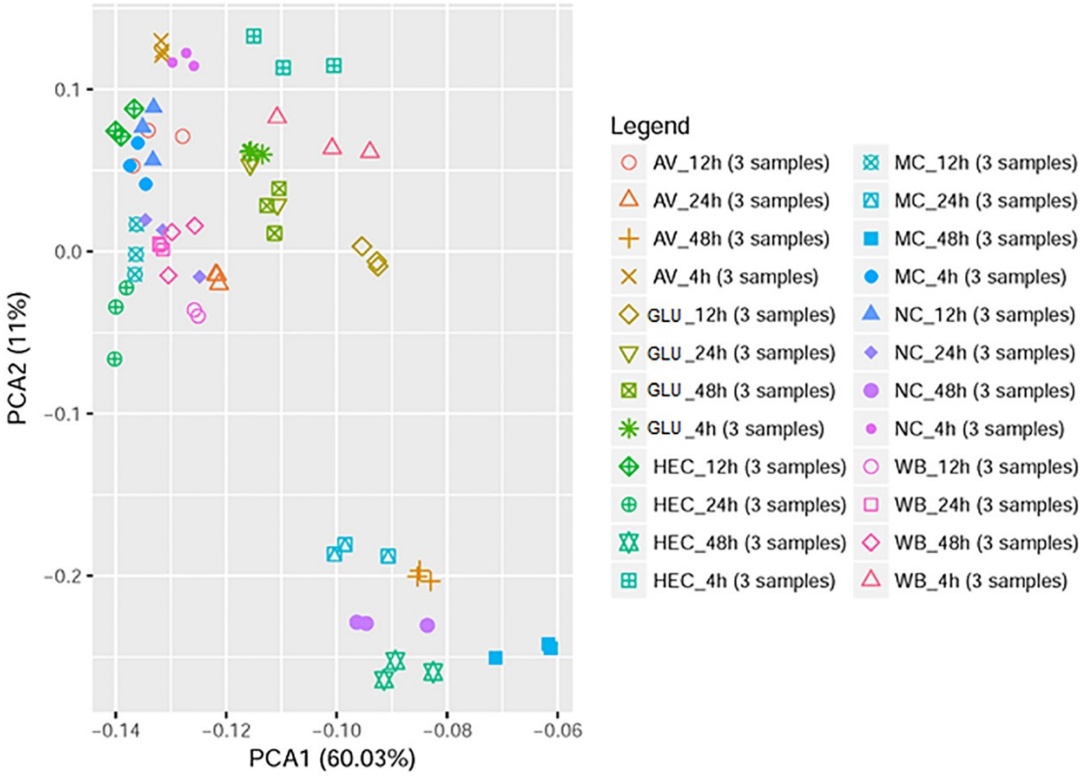

Supplementary Figure S5. Principal component analysis of transcriptomes from *P. oxalicum* strain Δ*PoxKu70* cultured on various carbon sources. AV: Avicel; WB: wheat bran; MC: methyl cellulose; HEC: 2-hydroxyethyl cellulose; GLU: glucose; NC: without carbon source. Principal component analysis (PCA) is a statistical procedure that uses an orthogonal transformation to convert a set of observations of possibly correlated variables into a set of values of linearly uncorrelated variables called principal components.

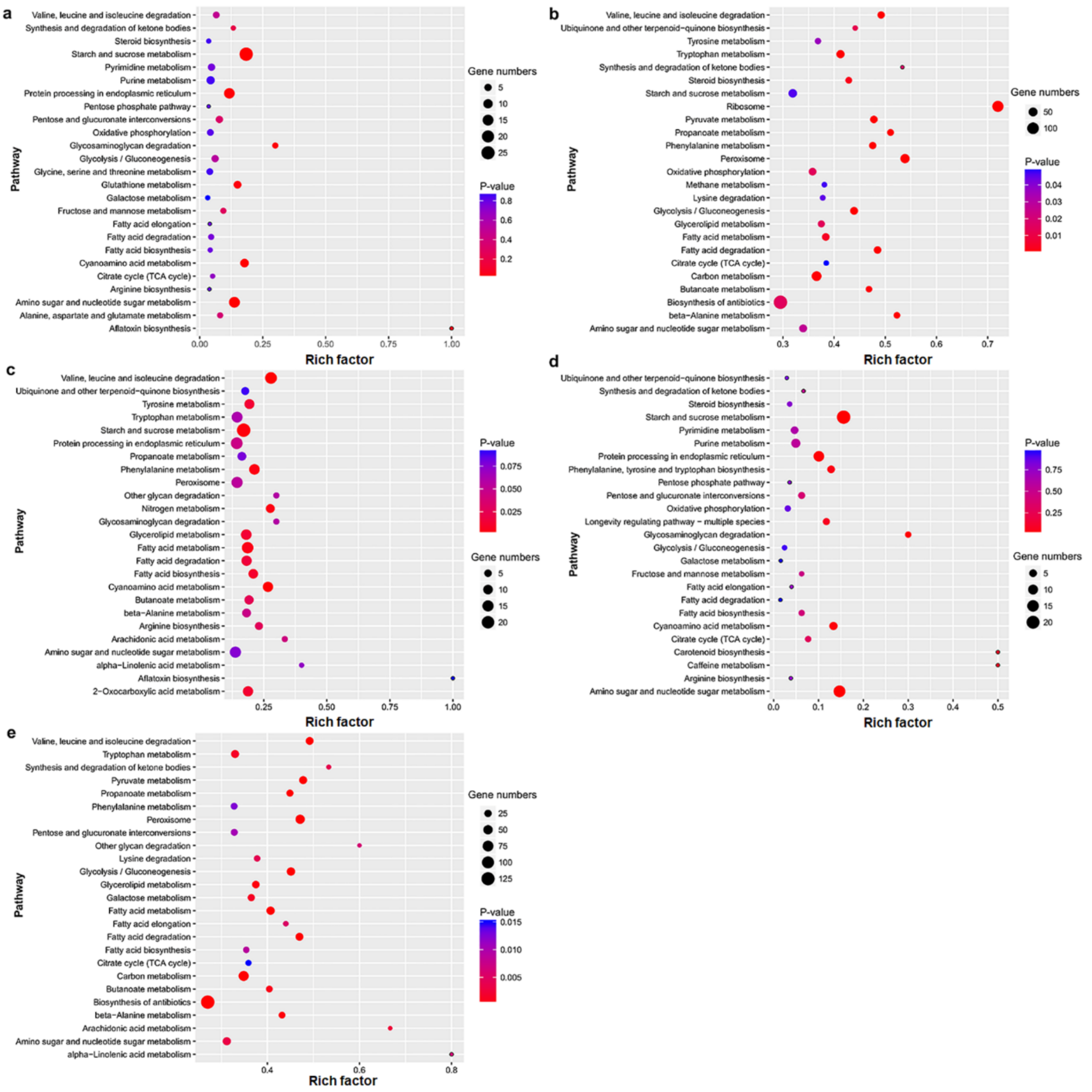

Supplementary Figure S6. Bubble diagram of DEGs at 4 h after induction as assessed by KEGG pathway enrichment. (a) AV vs NC; (b) WB vs NC; (c) MC vs NC; (d) HEC vs NC; (d) GLU vs NC. DEGs: differentially expressed genes; AV: Avicel; MC: methyl cellulose; HEC: 2-hydroxyethyl cellulose; GLU: glucose; NC: without carbon source. Rich factor means the value of enrichment factor that is the quotient of foreground value (the number of DEGs) and background value (total gene number).

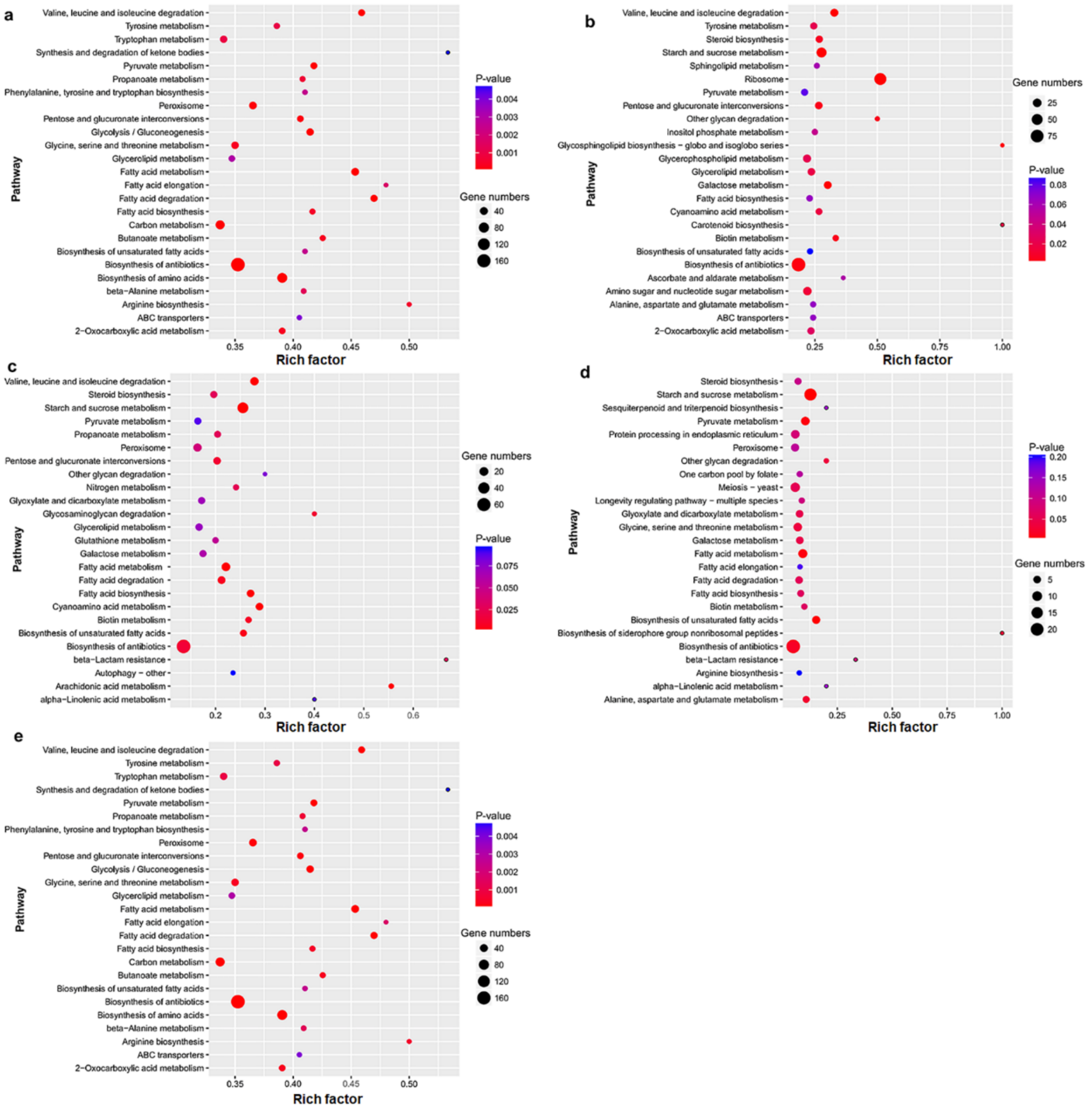

Supplementary Figure S7. Bubble diagram of DEGs at 12 h after induction as assessed by KEGG pathway enrichment. (a) AV vs NC; (b) WB vs NC; (c) MC vs NC; (d) HEC vs NC; (d) GLU vs NC. DEGs: differentially expressed genes; AV: Avicel; MC: methyl cellulose; HEC: 2-hydroxyethyl cellulose; GLU: glucose; NC: without carbon source. Rich factor means the value of enrichment factor that is the quotient of foreground value (the number of DEGs) and background value (total gene number).

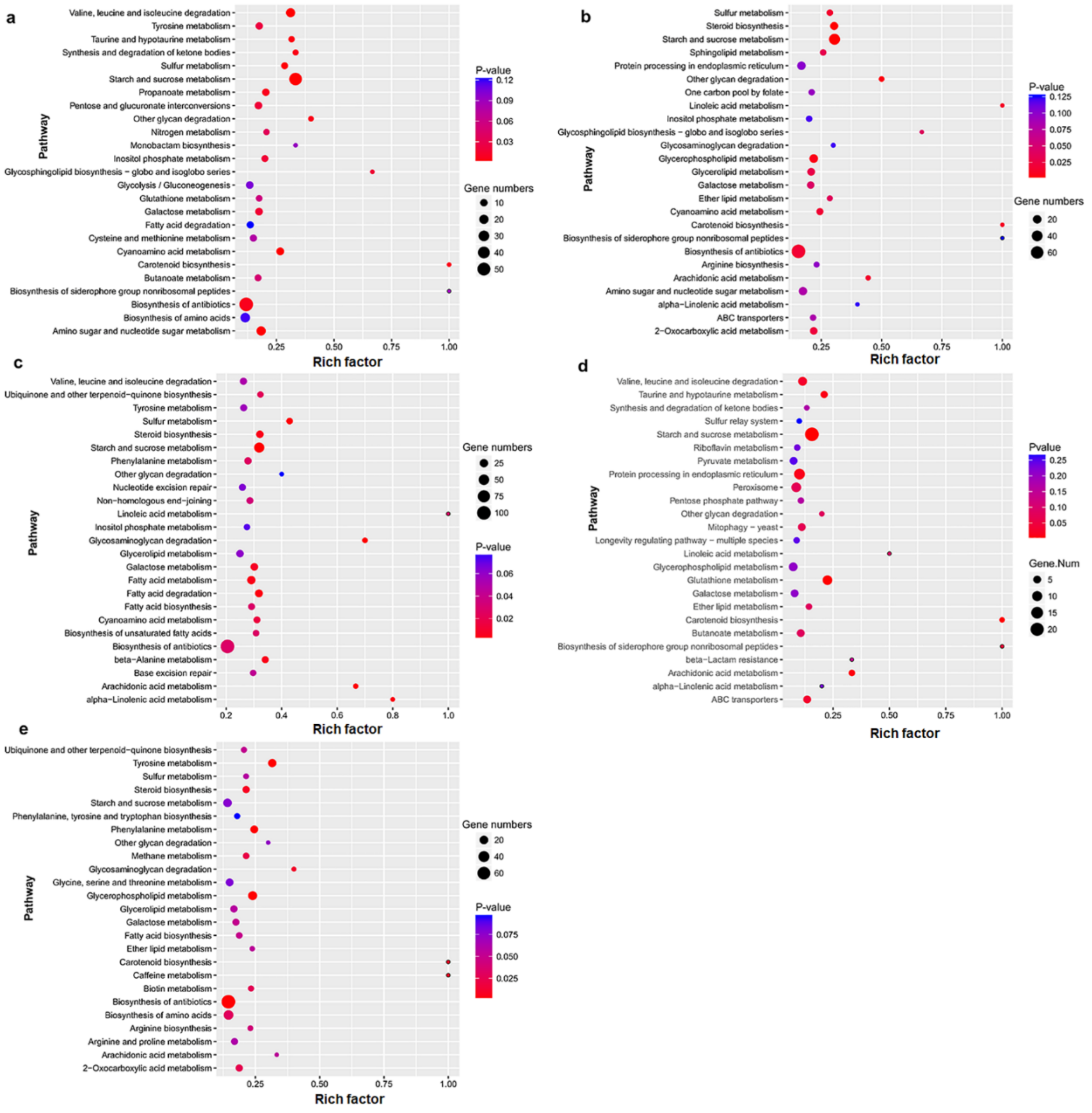

Supplementary Figure S8. Bubble diagram of differentially expressed genes at 24 h after induction as assessed by KEGG pathway enrichment. (a) AV vs NC; (b) WB vs NC; (c) MC vs NC; (d) HEC vs NC; (d) GLU vs NC. AV: Avicel; MC: methyl cellulose; HEC: 2-hydroxyethyl cellulose; GLU: glucose; NC: without carbon source. Rich factor means the value of enrichment factor that is the quotient of foreground value (the number of DEGs) and background value (total gene number).

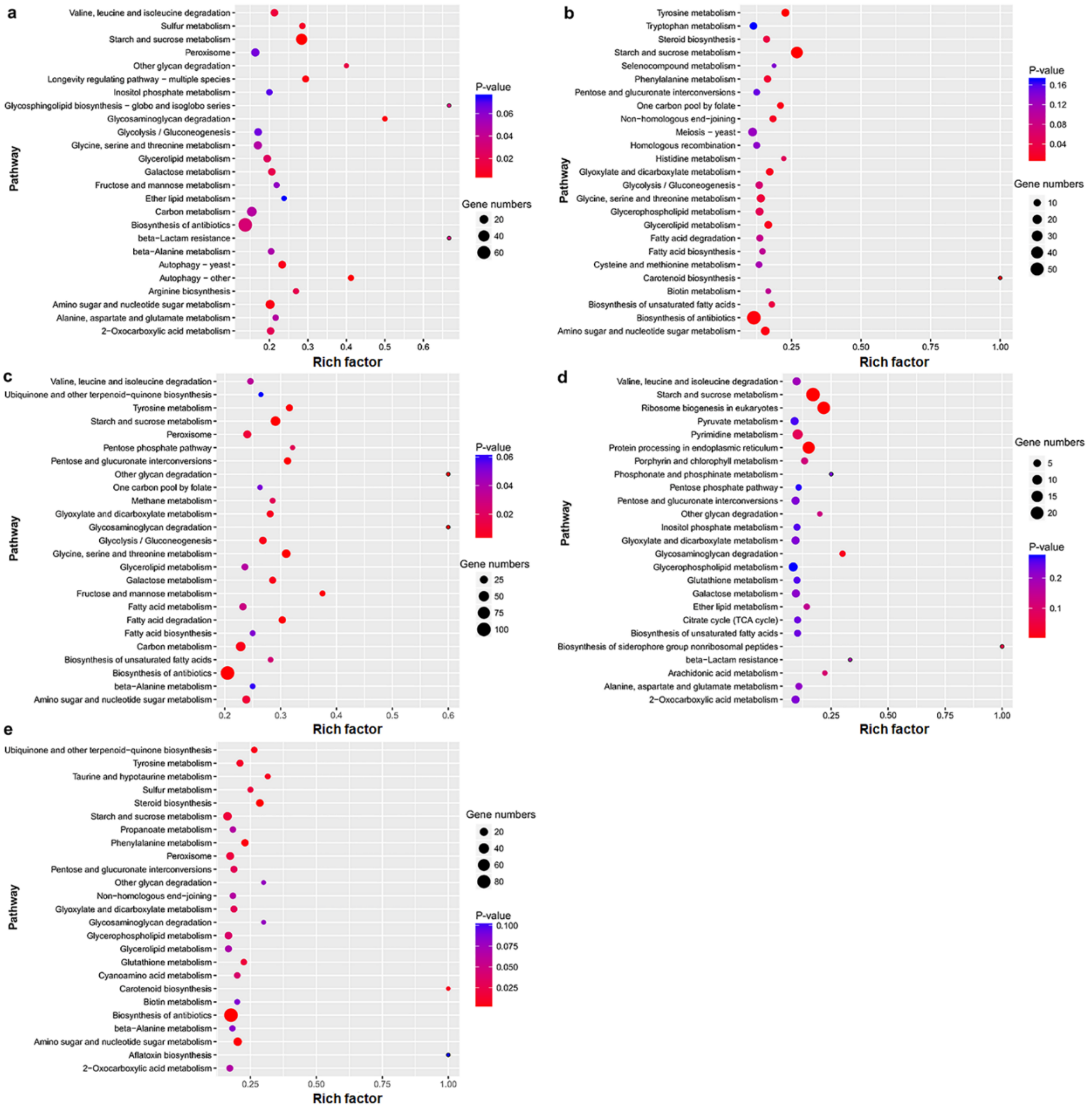

Supplementary Figure S9. Bubble diagram of DEGs at 48 h after induction as assessed by KEGG pathway enrichment. (a) AV vs NC; (b) WB vs NC; (c) MC vs NC; (d) HEC vs NC; (d) GLU vs NC. DEGs: differentially expressed genes; AV: Avicel; MC: methyl cellulose; HEC: 2-hydroxyethyl cellulose; GLU: glucose; NC: without carbon source. Rich factor means the value of enrichment factor that is the quotient of foreground value (the number of DEGs) and background value (total gene number).

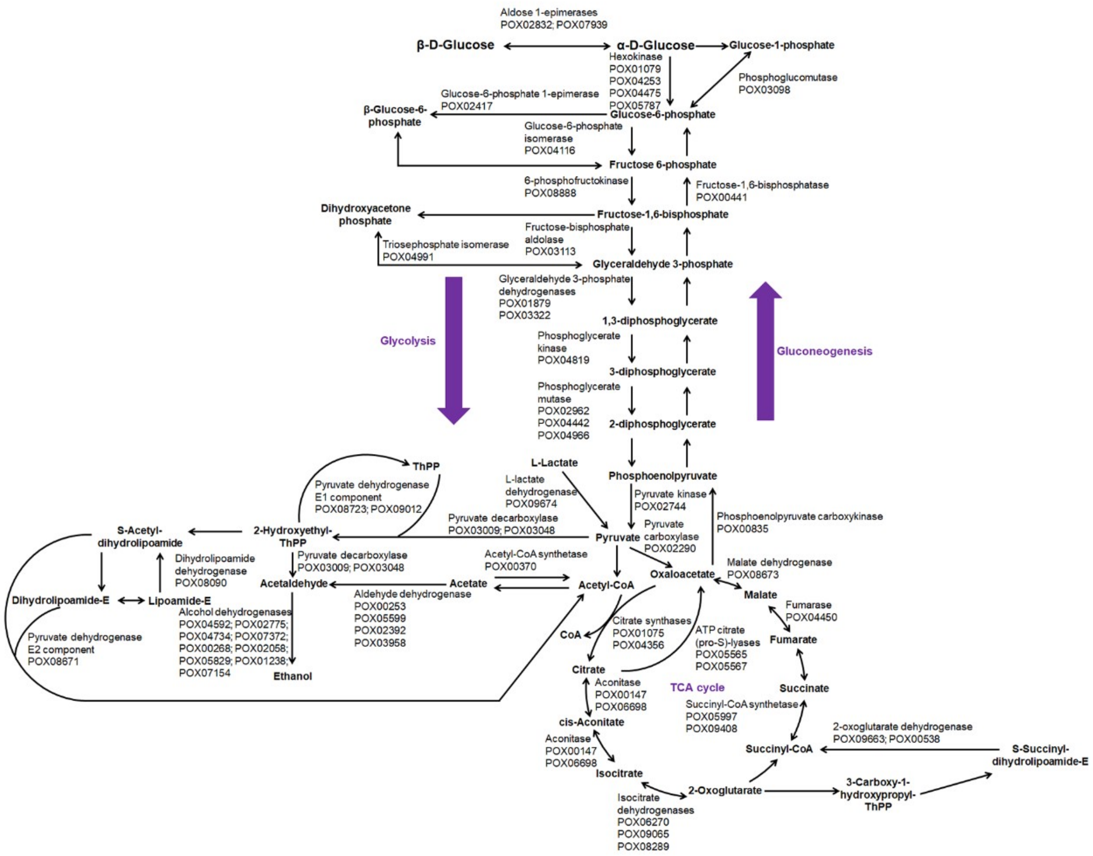

Supplementary Figure S10. Differentially expressed genes detected to be involved in glycolysis/gluconeogenesis and tricarboxylic acid (TCA) cycle.

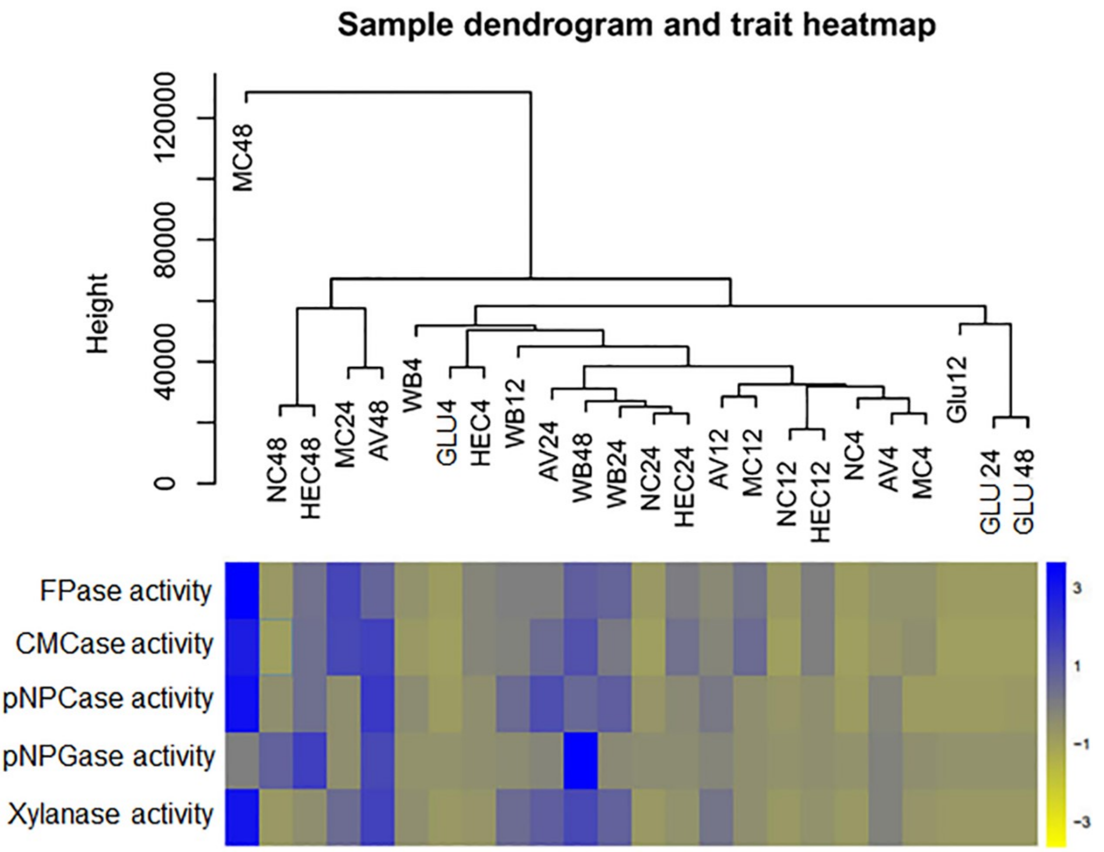

Supplementary Figure S11. Sample outlier detection. The heatmap shows different cellulase and xylanase production levels of *P. oxalicum* strain Δ*PoxKu70* cultured on various carbon sources for 4–48 h. AV: Avicel; WB: wheat bran; MC: methyl cellulose; HEC: 2-hydroxyethyl cellulose; GLU: glucose; NC: without carbon source. FPase: filter-paper cellulase, CMCase: carboxymethylcellulase, pNPCase: *p*-nitrophenyl-β-cellobiosidase, pNPGase: *p*-nitrophenyl-β-glucopyranosidase.

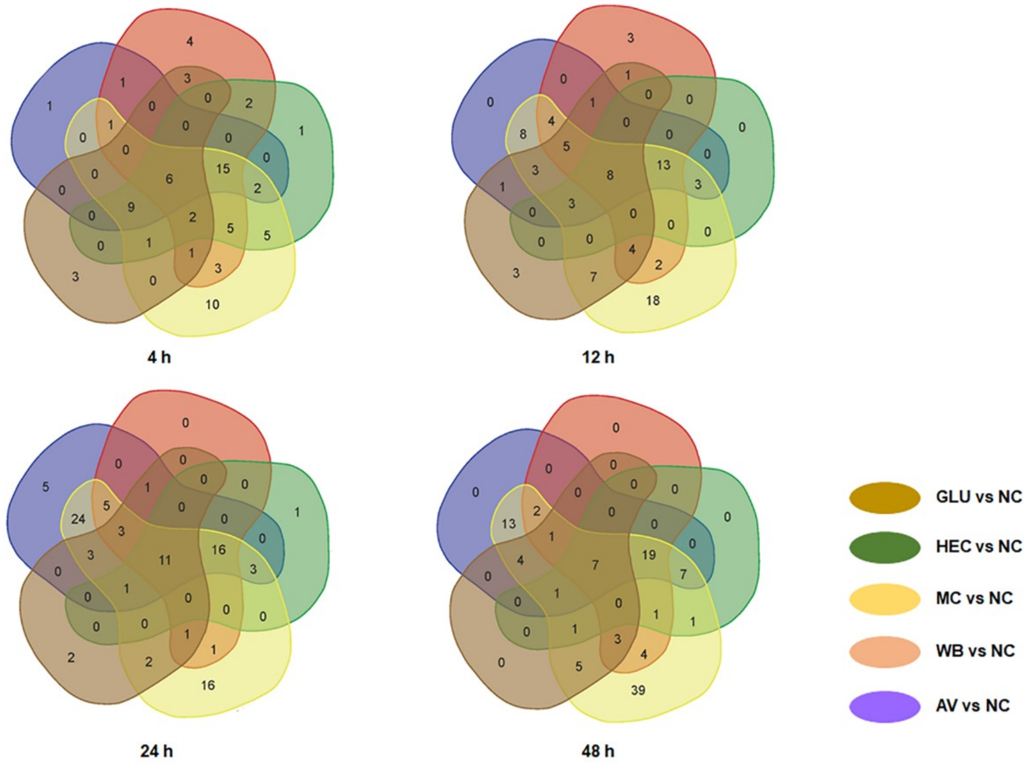

Supplementary Figure S12. Venn diagram indicating numbers of unique and shared differentially expressed genes in module MEivory from *P. oxalicum* in the presence of different carbon sources (AV, MC, HEC and WB) compared with NC. AV: Avicel; MC: methyl cellulose; HEC: 2-hydroxyethyl cellulose; GLU: glucose; NC: without carbon source.

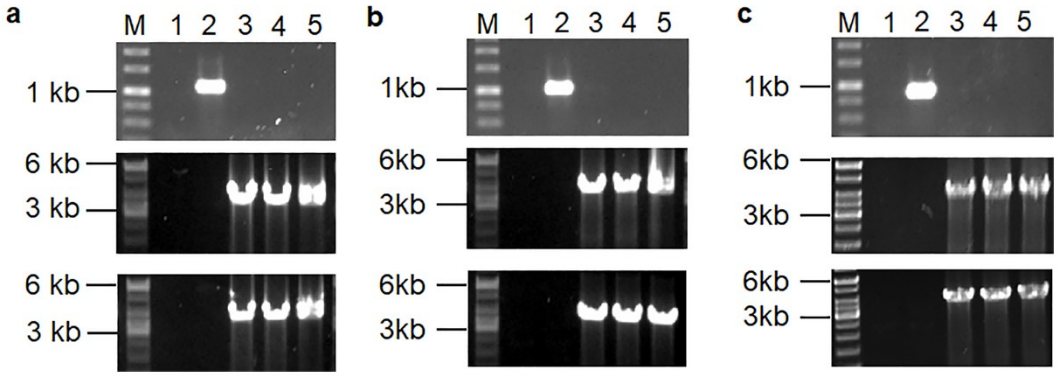

Supplementary Figure S13. PCR verification of mutants. (a) PCR validation of the *POX01118* mutant. From top to bottom, the pictures are the target gene validation diagram (the primers 1118F/R), the left cross validation diagram (1118UF/G418R) and the right cross validation diagram (G418F/1118DR). M: Marker; 1 line and 2 line represent negative control and positive control respectively; 3, 4 and 5 represent three randomly selected mutants, respectively. (b) PCR validation of Mutant *POX01474*. From top to bottom, the pictures are the target gene validation diagram (the primers 1474F/R), the left cross validation diagram (1474UF/G418R) and the right cross validation diagram (G418F/1474DR). M: Marker; 1 line and 2 line represent negative control and positive control respectively; 3, 4 and 5 represent three randomly selected mutants, respectively. (c) PCR validation of Mutant *POX01678*. From top to bottom, the pictures are the target gene validation diagram (the primers 1678F/R), the left cross validation diagram (1678UF/G418R) and the right cross validation diagram (G418F/1118DR). M: Marker; 1 line and 2 line represent negative control and positive control respectively; 3, 4 and 5 represent three randomly selected mutants, respectively.

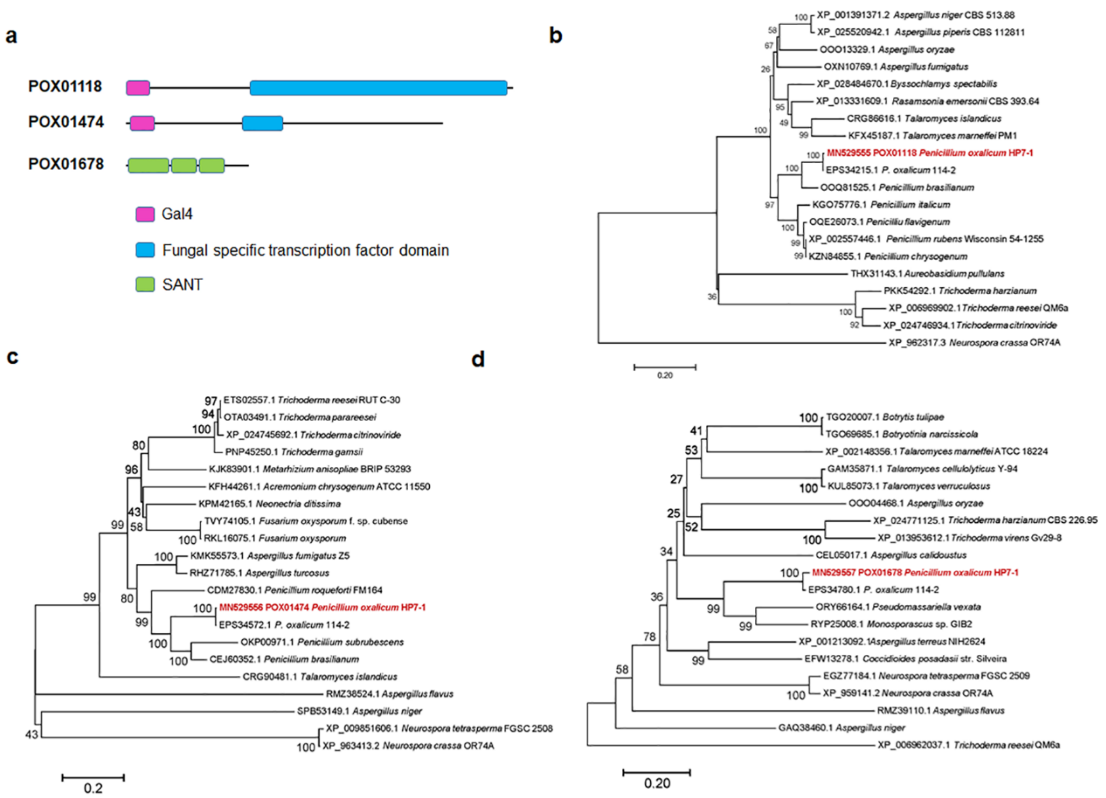

Supplementary Figure S14. Sequence analysis (a) of the proteins POX01118, POX01474 and POX01678, and their phylogenetic analysis (c-d). SANT: 'SANT, SWI3, ADA2, N-CoR and TFIIIB' DNA-binding domain. The phylogenetic trees are constructed based on the neighbor-joining method and Poisson model. Bootstrap values are shown at nodes, derived from 1000 replicates.
