# Supplementary material for: Weighted Gene Co-expression Network Analysis Identifies Critical Genes for the Production of Cellulase and Xylanase in Penicillium oxalicum
Source: Front Microbiol. 2020 Mar 27;11:520. doi: 10.3389/fmicb.2020.00520 (PMC7118919; doi:10.3389/fmicb.2020.00520)
Supplement: Supplementary file 2 [file Table_1.DOCX]

**Supplementary Table S1**. Primers used for mutant construction.

| **Primer Names** | **Sequences (5’-3’)** |
| --- | --- |
| 1118UF | GTTCGGGTTTCACTCTAATGG |
| 1118UR | CTTCAATATCATCTTCTGTCGAAAAGCGACGGCAAGACAT |
| 1118DF | TTTAGAGGTAATCCTTCTTTCTAGGCCCCCAGCTTCATAAATA |
| 1118DR | GAGAATCCGAACATGAGCAA |
| 1118NF | AGGTATTCCACCCATTAGG |
| 1118NF | CGAGTCTTGGTGTGATAAACTG |
| 1118F | GCCTGGCTTATGGTATGTGC |
| 1118R | CGCACGGCTTGAATGTATCT |
| 1678UF | CGATTCAAAATGGTAATGCTA |
| 1678UR | CTTCAATATCATCTTCTGTCGATGAGTAAACTGTGAGTGCAGG |
| 1678DF | TTTAGAGGTAATCCTTCTTTCTAGGACATCCTGATGGACTCGAA |
| 1678DR | CAAAAGATTGACGACCGAT |
| 1678NF | AGGGAGTGGTCCAGGTATGT |
| 1678NR | GCTGGCTGGTAAGACCTAAC |
| 1474F | TTCACCAGCGACCATTCATA |
| 1474R | GAGCATTTGCCAGAAGTTGA |
| 1474UF | AGTCCATCATTCTGCCTTCC |
| 1474UR | CTTCAATATCATCTTCTGTCGAACATCGAGAGTGTGAGAGCC |
| 1474DF | TTTAGAGGTAATCCTTCTTTCTAGAGAAAATAAAGATAACGCTGACT |
| 1474DR | CTCAAAAGGGTCATCAAGGT |
| 1474NF | TGCCACGGCTTCCTACCAAA |
| 1474NR | AGCCTTGCTGGGCGTGTTTT |
| 1678F | GAGAAAGACGATGGTGGACA |
| 1678R | ACTCGCACTCAGCACTACC |
| G418F | TCGACAGAAGATGATATTGAAG |
| G418R | CTAGAAAGAAGGATTACCTCTAAA |
