# Supplementary material for: Weighted Gene Co-expression Network Analysis Identifies Critical Genes for the Production of Cellulase and Xylanase in Penicillium oxalicum
Source: Front Microbiol. 2020 Mar 27;11:520. doi: 10.3389/fmicb.2020.00520 (PMC7118919; doi:10.3389/fmicb.2020.00520)
Supplement: Supplementary file 3 [file Table_2.DOCX]

**Supplementary Table S2. List of 23 chosen carbon sources.**

| **Number** | **Names of carbon sources** |
| --- | --- |
| 1 | Sorbitol |
| 2 | Lactose |
| 3 | Mannitol |
| 4 | Carboxymethylcellulose (CMC) |
| 5 | Fructose |
| 6 | Cellobiose |
| 7 | 2-Hydroxyethyl cellulose (HEC) |
| 8 | Maltose |
| 9 | Galactose |
| 10 | Methyl cellulose (MC) |
| 11 | Dextrin |
| 12 | α-cellulose |
| 13 | Xylose |
| 14 | Mannose |
| 15 | Glycerol |
| 16 | Sucrose |
| 17 | Avicel (AV) |
| 18 | Glucose (GLU) |
| 19 | Wheat bran (WB) |
| 20 | D-arabinose |
| 21 | L-arabinose |
| 22 | Rhamnose |
| 23 | Sigmacell cellulose (SC50) |
| 24 | Without carbon source (NC) |
