# Supplementary material for: Weighted Gene Co-expression Network Analysis Identifies Critical Genes for the Production of Cellulase and Xylanase in Penicillium oxalicum
Source: Front Microbiol. 2020 Mar 27;11:520. doi: 10.3389/fmicb.2020.00520 (PMC7118919; doi:10.3389/fmicb.2020.00520)
Supplement: Supplementary file 4 [file Table_3.DOCX]

**Supplementary Table S3** Summary of transcriptomes from *P. oxalicum* strain ∆*PoxKu70* cultured on various carbon sources.

| **Number** | **Clean reads** | **Genome mapping rate** | **Gene map Rate** | **Culture conditions** |
| --- | --- | --- | --- | --- |
| 1 | 21909744 | 98.65% | 70.84 | Avicel, 4h |
| 2 | 21911236 | 98.60% | 70.68 |  |
| 3 | 21863242 | 98.52% | 70.86 |  |
| 4 | 21885854 | 98.03% | 68.41 | Avicel, 12h |
| 5 | 21880489 | 98.18% | 68.2 |  |
| 6 | 21885732 | 98.06% | 67.27 |  |
| 7 | 21905427 | 98.42% | 68.69 | Avicel, 24h |
| 8 | 21905096 | 98.20% | 67.7 |  |
| 9 | 21899283 | 98.23% | 68.23 |  |
| 10 | 21592247 | 98.42% | 67.13 | Avicel, 48h |
| 11 | 21627048 | 98.25% | 67.88 |  |
| 12 | 21522553 | 98.36% | 67.78 |  |
| 13 | 21903582 | 98.47% | 69.75 | Glucose, 4h |
| 14 | 21904254 | 98.47% | 69.6 |  |
| 15 | 21899612 | 98.37% | 70.38 |  |
| 16 | 21746161 | 98.12% | 68.05 | Glucose, 12h |
| 17 | 19167016 | 98.20% | 67.8 |  |
| 18 | 18683473 | 98.12% | 67.67 |  |
| 19 | 18927492 | 98.10% | 67.25 | Glucose, 24h |
| 20 | 19137915 | 98.03% | 67.54 |  |
| 21 | 21438203 | 98.05% | 66.18 |  |
| 22 | 18966651 | 98.11% | 67.16 | Glucose, 48h |
| 23 | 20381782 | 98.33% | 67.21 |  |
| 24 | 21750850 | 98.54% | 67.82 |  |
| 25 | 21905265 | 98.58% | 71.72 | 2-Hydroxyetgyl cellulose, 4h |
| 26 | 21909595 | 98.47% | 72.07 |  |
| 27 | 21877277 | 98.54% | 72.94 |  |
| 28 | 21859788 | 98.13% | 67.94 | 2-Hydroxyetgyl cellulose, 12h |
| 29 | 21882132 | 98.13% | 68.02 |  |
| 30 | 21882350 | 98.12% | 68.7 |  |
| 31 | 21904406 | 98.18% | 66.57 | 2-Hydroxyetgyl cellulose, 24h |
| 32 | 21896104 | 97.90% | 65.98 |  |
| 33 | 21897096 | 98.01% | 67.01 |  |
| 34 | 21744502 | 98.29% | 66.47 | 2-Hydroxyetgyl cellulose, 48h |
| 35 | 21645068 | 98.19% | 64.62 |  |
| 36 | 18234262 | 98.01% | 61.95 |  |
| 37 | 21902916 | 98.49% | 70.72 | Methyl cellulose, 4h |
| 38 | 21903452 | 98.43% | 65.82 |  |
| 39 | 21904700 | 98.56% | 69.03 |  |
| 40 | 21875666 | 98.17% | 67.61 | Methyl cellulose, 12h |
| 41 | 21884665 | 98.05% | 68.48 |  |
| 42 | 21875927 | 98.08% | 67.73 |  |
| 43 | 21894433 | 97.84% | 66.76 | Methyl cellulose, 24h |
| 44 | 21904846 | 97.92% | 67.03 |  |
| 45 | 21899392 | 97.80% | 66.72 |  |
| 46 | 21640164 | 98.31% | 62.2 | Methyl cellulose, 48h |
| 47 | 18866593 | 98.18% | 64.49 |  |
| 48 | 21679647 | 98.31% | 63.09 |  |
| 49 | 21902120 | 98.48% | 70.54 | No carbon, 4h |
| 50 | 21905534 | 98.59% | 70.85 |  |
| 51 | 21910358 | 98.60% | 70.65 |  |
| 52 | 21906028 | 98.34% | 68.6 | No carbon, 12h |
| 53 | 21880940 | 98.19% | 67.65 |  |
| 54 | 21870058 | 98.10% | 68.11 |  |
| 55 | 21737049 | 98.04% | 67.06 | No carbon, 24h |
| 56 | 21873418 | 98.28% | 66.02 |  |
| 57 | 21886329 | 98.26% | 66.02 |  |
| 58 | 21906013 | 98.38% | 62.93 | No carbon, 48h |
| 59 | 21650545 | 98.05% | 64.65 |  |
| 60 | 21886660 | 98.08% | 64.43 |  |
| 61 | 21907573 | 98.23% | 68.05 | Wheat bran, 4h |
| 62 | 21904260 | 98.64% | 68.08 |  |
| 63 | 21900697 | 98.50% | 68.48 |  |
| 64 | 21207458 | 98.09% | 66.64 | Wheat bran, 12h |
| 65 | 21896960 | 98.42% | 68.41 |  |
| 66 | 20360669 | 98.32% | 68.72 |  |
| 67 | 21882626 | 97.92% | 67.7 | Wheat bran, 24h |
| 68 | 21882060 | 98.00% | 67.3 |  |
| 69 | 21884233 | 98.07% | 67.83 |  |
| 70 | 21903519 | 98.03% | 67.93 | Wheat bran, 48h |
| 71 | 21907857 | 98.08% | 68.23 |  |
| 72 | 21904642 | 98.10% | 67.64 |  |
