# Supplementary material for: Weighted Gene Co-expression Network Analysis Identifies Critical Genes for the Production of Cellulase and Xylanase in Penicillium oxalicum
Source: Front Microbiol. 2020 Mar 27;11:520. doi: 10.3389/fmicb.2020.00520 (PMC7118919; doi:10.3389/fmicb.2020.00520)
Supplement: Supplementary file 8 [file Table_7.DOCX]

**Supplementary Table S7. Summary of number of common DEGs altered in response to different carbon sources at transcriptional level.**

| **Induction time** | **Proteins encoded by DEGs** | **Description of number of common DEGs altered at transcriptional level** | **AV** | **WB** | **MC** | **HEC** | **GLU** |
| --- | --- | --- | --- | --- | --- | --- | --- |
| **4 h** | CAZymes | Total number of DEGs | 66 | 224 | 51 | 43 | 187 |
|  |  | Number of DEGs being specific to carbon source | 0 | 66 | 6 | 4 | 46 |
|  |  | Number of DEGs being response to all carbon sources | 15 | | | | |
|  | TFs | Total number of DEGs | 62 | 175 | 36 | 34 | 136 |
|  |  | Number of DEGs being specific to carbon source | 2 | 56 | 1 | 4 | 20 |
|  |  | Number of DEGs being response to all carbon sources | 13 | | | | |
|  | Transporters | Total number of DEGs | 39 | 145 | 63 | 31 | 135 |
|  |  | Number of DEGs being specific to carbon source | 0 | 42 | 3 | 1 | 31 |
|  |  | Number of DEGs being response to all carbon sources | 11 | | | | |
|  | GPCRs | Total number of DEGs | 0 | 12 | 1 | 1 | 4 |
|  |  | Number of DEGs being specific to carbon source | 0 | 7 | 0 | 0 | 0 |
|  |  | Number of DEGs being response to all carbon sources | 0 | | | | |
| **12h** | CAZymes | Total number of DEGs | 68 | 139 | 107 | 37 | 183 |
|  |  | Number of DEGs being specific to carbon source | 2 | 28 | 12 | 0 | 68 |
|  |  | Number of DEGs being response to all carbon sources | 10 | | | | |
|  | TFs | Total number of DEGs | 41 | 88 | 61 | 24 | 131 |
|  |  | Number of DEGs being specific to carbon source | 5 | 15 | 4 | 1 | 54 |
|  |  | Number of DEGs being response to all carbon sources | 5 | | | | |
|  | Transporters | Total number of DEGs | 55 | 88 | 69 | 26 | 125 |
|  |  | Number of DEGs being specific to carbon source | 6 | 18 | 9 | 0 | 51 |
|  |  | Number of DEGs being response to all carbon sources | 8 | | | | |
|  | GPCRs | Total number of DEGs | 0 | 7 | 0 | 1 | 7 |
|  |  | Number of DEGs being specific to carbon source | 0 | 2 | 0 | 0 | 2 |
|  |  | Number of DEGs being response to all carbon sources | 0 | | | | |
| **24 h** | CAZymes | Total number of DEGs | 102 | 132 | 148 | 52 | 132 |
|  |  | Number of DEGs being specific to carbon source | 7 | 19 | 33 | 3 | 34 |
|  |  | Number of DEGs being response to all carbon sources | 7 | | | | |
|  | TFs | Total number of DEGs | 72 | 80 | 88 | 31 | 99 |
|  |  | Number of DEGs being specific to carbon source | 13 | 14 | 21 | 3 | 32 |
|  |  | Number of DEGs being response to all carbon sources |  | | | | |
|  | Transporters | Total number of DEGs | 69 | 92 | 99 | 36 | 77 |
|  |  | Number of DEGs being specific to carbon source | 12 | 15 | 24 | 2 | 22 |
|  |  | Number of DEGs being response to all carbon sources | 10 | | | | |
|  | GPCRs | Total number of DEGs | 3 | 4 | 3 | 1 | 5 |
|  |  | Number of DEGs being specific to carbon source | 0 | 1 | 1 | 0 | 1 |
|  |  | Number of DEGs being response to all carbon sources | 0 | | | | |
| **48 h** | CAZymes | Total number of DEGs | 137 | 101 | 128 | 57 | 118 |
|  |  | Number of DEGs being specific to carbon source | 24 | 14 | 22 | 3 | 20 |
|  |  | Number of DEGs being response to all carbon sources | 6 | | | | |
|  | TFs | Total number of DEGs | 78 | 76 | 83 | 34 | 82 |
|  |  | Number of DEGs being specific to carbon source | 11 | 16 | 20 | 3 | 20 |
|  |  | Number of DEGs being response to all carbon sources | 7 | | | | |
|  | Transporters | Total number of DEGs | 90 | 76 | 91 | 40 | 74 |
|  |  | Number of DEGs being specific to carbon source | 8 | 19 | 15 | 2 | 20 |
|  |  | Number of DEGs being response to all carbon sources | 6 | | | | |
|  | GPCRs | Total number of DEGs | 2 | 1 | 3 | 0 | 2 |
|  |  | Number of DEGs being specific to carbon source | 1 | 0 | 2 | 0 | 1 |
|  |  | Number of DEGs being response to all carbon sources | 0 | | | | |

Note: TF: transcription factor; CAZymes: carbohydrate-active enzymes; GPCR: G protein-coupled receptor; AV: Avicel; WB: wheat bran; MC: methyl cellulose; HEC: 2-hydroxyethyl cellulose; GLU: glucose. DEG: differentially expressed gene.
